# A new vesicle trafficking regulator CTL1 plays a crucial role in ion homeostasis

**DOI:** 10.1371/journal.pbio.2002978

**Published:** 2017-12-28

**Authors:** Yi-Qun Gao, Jiu-Geng Chen, Zi-Ru Chen, Dong An, Qiao-Yan Lv, Mei-Ling Han, Ya-Ling Wang, David E. Salt, Dai-Yin Chao

**Affiliations:** 1 National Key Laboratory of Plant Molecular Genetics, Chinese Academy of Sciences Center for Excellence in Molecular Plant Sciences, Institute of Plant Physiology and Ecology, Shanghai Institutes for Biological Sciences, Chinese Academy of Sciences, Shanghai, China; 2 University of Chinese Academy of Sciences, Beijing, China; 3 Centre for Plant Integrative Biology, School of Biosciences, University of Nottingham, Sutton Bonington Campus, Loughborough, United Kingdom; University of California San Diego, United States of America

## Abstract

Ion homeostasis is essential for plant growth and environmental adaptation, and maintaining ion homeostasis requires the precise regulation of various ion transporters, as well as correct root patterning. However, the mechanisms underlying these processes remain largely elusive. Here, we reported that a choline transporter gene, *CTL1*, controls ionome homeostasis by regulating the secretory trafficking of proteins required for plasmodesmata (PD) development, as well as the transport of some ion transporters. Map-based cloning studies revealed that *CTL1* mutations alter the ion profile of *Arabidopsis thaliana*. We found that the phenotypes associated with these mutations are caused by a combination of PD defects and ion transporter misregulation. We also established that *CTL1* is involved in regulating vesicle trafficking and is thus required for the trafficking of proteins essential for ion transport and PD development. Characterizing choline transporter-like 1 (CTL1) as a new regulator of protein sorting may enable researchers to understand not only ion homeostasis in plants but also vesicle trafficking in general.

## Author summary

Ion transporters play a key role in mineral nutrients uptake and transport of plants. Their cellular and subcellular localization is essential for fulfilling their functions and are therefore generally fine-tuned. However, the molecular mechanisms underlying this process remain largely unclear. In this study, we analyze the role of the choline transporter CTL1 in *A*. *thaliana* and find that it controls dynamic cell trafficking, a fundamental process that plays vital roles in cell signaling, development, and protein sorting. We also show that CTL1 regulates the expression pattern of different ion transporters through the modulation of vesicle trafficking. These results suggest that CTL1 is a new component of the vesicle trafficking machinery that is also required for ion homeostasis, which link these processes and shed light on the underlying molecular mechanisms.

## Introduction

The root is highly specified and well organized for the uptake and transport of water and mineral nutrients from the highly heterogeneous soil environment. Plants have evolved complex gene networks to fine tune the activities of different transporters, as well as the functions of different cell wall structures and processes associated with cell-to-cell communication, to match the nutrients supplied by the soil with the normal growth- and development-related needs of their cells. These processes are regulated at both the transcriptional and posttranscriptional levels. At the transcriptional level, the expression of transporters and genes that function in root patterning and development is orchestrated by a series of transcription factors. Ectopic expression of a transporter may result in disorders of ion homeostasis. For example, enhanced expression of *A*. *thaliana* high affinity K+ transporter 1 (*AtHKT1*), specifically in the stele leads to reductions in shoot sodium ion (Na^+^) concentrations; however, constitutive overexpression of *AtHKT1* throughout the plant leads to significant increases in shoot Na^+^ concentrations [1].

At the posttranscriptional level, transporters can be regulated by processes such as protein modification, degradation, and trafficking. For example, the subcellular localization of iron regulated transporter 1 (IRT1), which transports bivalent cations including iron (Fe^2+^), zinc (Zn^2+^), manganese (Mn^2+^), cobalt (Co^2+^), and cadmium (Cd^2+^) ions, is mediated by monoubiquitin- and clathrin-dependent endocytosis involving the IRT1 degradation factor1 RING-type (IDF1 RING) E3 ligases [2,3]. FYVE domain protein required for endosomal sorting 1 (FYVE1), a phosphatidylinositol-3-phosphate (PI3P)-binding protein, interacts with IRT1 and mediates the recycling of IRT1 from the endosome back to the plasma membrane (PM) [4], suggesting that membrane lipids and lipid-binding proteins play important roles in protein trafficking and ion homeostasis.

Phosphatidylinositol (PI) sphingolipids and phosphatidic acids (PAs) have all been found to participate in the regulation of vesicle trafficking [5,6]. However, as the most abundant phospholipids in the PM of eukaryotic cells [7,8], phosphatidylcholines (PCs) have not been shown to be involved in either cargo trafficking or protein subcellular localization, although they have been found to play a crucial role in signal transduction [9]. These important phospholipids incorporate choline as a head group and can be used as a substrate to produce choline in reactions catalyzed by phospholipase D (PLD). In animals, choline is an essential nutrient, because it serves not only as a component of membrane lipids and lipoprotein but also serves as a precursor of many essential molecules such as the neurotransmitter acetylcholine and the osmoregulator betaine [10]. The choline transporter is required for choline homeostasis, as well as secretion of acetylcholine [11–13]. Choline is also produced in plants, but its biological function was rarely studied. A recent study showed that the trans-Golgi network (TGN)-localized choline transporter choline transporter-like 1 (CTL1) is involved in the formation of the phloem sieve plate and sieve pore; however, the mechanism underlying its role in these processes remains unknown [14].

Here, we identified a new *CTL1* allele by screening ethyl methanesulfonate (EMS)-mutagenized plants to identify those with an altered leaf ionome. Our results showed that CTL1 is required for vesicle trafficking and that *CTL1* mutations result in the disruption of plasmodesmata (PD) and the normal localization of PM proteins, including ion transporters and PD callose-binding (PDCB) proteins.

## Results

### Identification of a novel ionomic mutant

**Fig 1:**
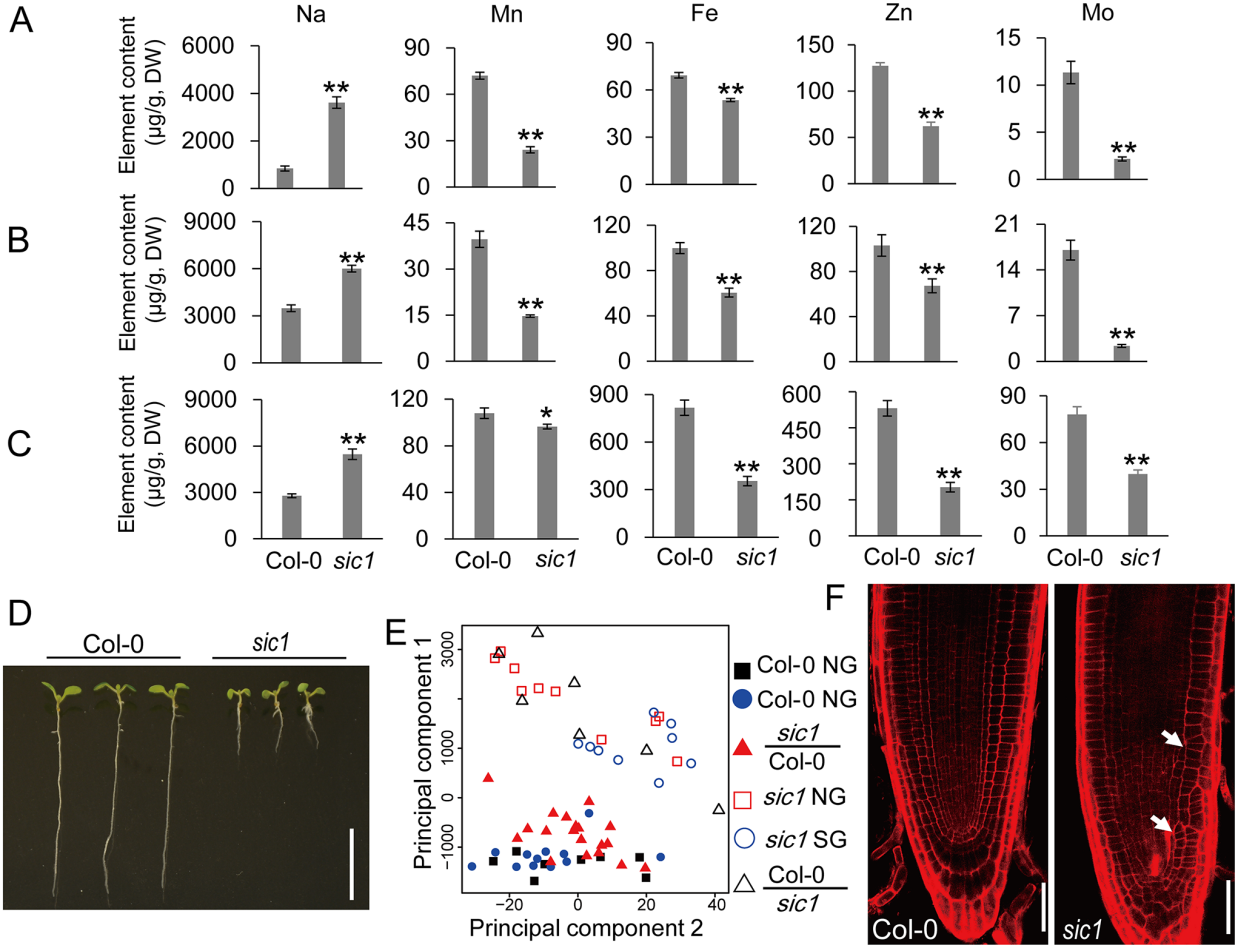
*sic1* is defective with respect to ion homeostasis and development. (A-C) The concentrations of Na, Mn, Fe, Zn, and Mo in the leaves (A-B) and roots (C) of Col-0 and *sic1* grown in artificial soil (A) or in hydroponics (B and C). The data represent the means ± SE; the asterisks above the bars represent a statistically significant difference (* indicates *p* < 0.05 and ** indicates *p* < 0.01) calculated using Student *t* test (*n* = 7–23). (D) Growth phenotype of 6-day-old Col-0 and *sic1* seedlings. The scale bar represents 1 cm. (E) PCA of the leaf ionome of 5-week-old *sic1* and Col-0 grafted plants. PCA was performed based on the leaf concentrations of Na, Mn, Fe, Zn, and Mo. *sic1*/Col-0, grafted plants with *sic1* shoots and Col-0 roots; Col-0/*sic1*, grafted plants with Col-0 shoots and *sic1* roots (*n* = 7–19). (F) Root patterning of Col-0 and *sic1* plants. The white arrow indicates irregular cell division in *sic1*. The scale bar represents 40 μm. All numerical data used in the generation of this figure can be found in S1 Data. Col-0, Columbia-0; DW, dry weight; Fe, iron; Mn, manganese, Mo, molybdenum; Na, sodium; NG, non-grafted plants; PCA, principle component analysis; SG, self-grafted Col-0; *sic1*, significant ionome changes 1; Zn, zinc.

A previous study screened fast neutron mutagenized *A*. *thaliana* plants for mutants with an altered leaf ionome to help unravel the gene networks regulating plant ion homeostasis [15], several of which have since been well characterized [16,17]. Using the same strategy, we isolated a new mutant that displays significantly increased leaf concentrations of Na and decreased leaf concentrations of Mn, Fe, Zn, and molybdenum (Mo) when grown in artificial soil (Fig 1A, S1 Table). We named this mutant *significant ionome changes 1* (*sic1*). The leaf ionomic phenotypes of *sic1* observed in plants grown in hydroponics were similar to those observed in plants grown in soil (Fig 1B); however, the mutant displayed significant alterations in the concentrations of more elements—as it displayed increases in lithium (Li), boron (B), and sulfur (S) concentrations and decreases in phosphorus (P), potassium (K), calcium (Ca), Co, nickel (Ni), and copper (Cu) concentrations (S2 Table)—compared with the wild-type plant. The hydroponically cultured root of *sic1* also displayed impaired ion homeostasis, as well as ionomic changes similar to those observed in *sic1* leaves (Fig 1C, S3 Table), suggesting that the ionomic changes characteristic of *sic1* may result from defects in ion uptake or short-distance transport in root.

In addition to exhibiting ionomics phenotypic abnormalities, *sic1* also exhibited clear developmental defects. Specifically, *sic1* displayed defects in leaf and root elongation (Fig 1D, S1 Fig) and had fewer leaves than its wild-type counterpart (S1 Fig); however, there was no significant difference in flowering time between the two plants. To test the hypothesis that the ionomic phenotypes of *sic1* resulted from defects in ion uptake or short-distance transport by the roots, we performed reciprocal grafting experiments involving wild-type and *sic1* plants. Principle component analysis (PCA) showed that the leaf ionomic phenotypes of grafted plants with a *sic1* scion and wild-type root were similar to those of nongrafted and self-grafted wild-type plants. However, grafted plants with a wild-type scion and *sic1* root had a leaf ionome that was indistinguishable from that of *sic1* (Fig 1E, S4 Table). Such evidence indicates that the leaf ionomic phenotype is driven by the root. Interestingly, the shoot developmental phenotypes of *sic1* were also partially driven by the root (S2 Fig). These data suggested that *sic1* root function is defective. Consistent with this finding, we also found that the cells in the root of *sic1* were organized in an irregular manner, probably as a result of irregular cell division (Fig 1F).

### Map-based cloning of *sic1*

**Fig 2:**
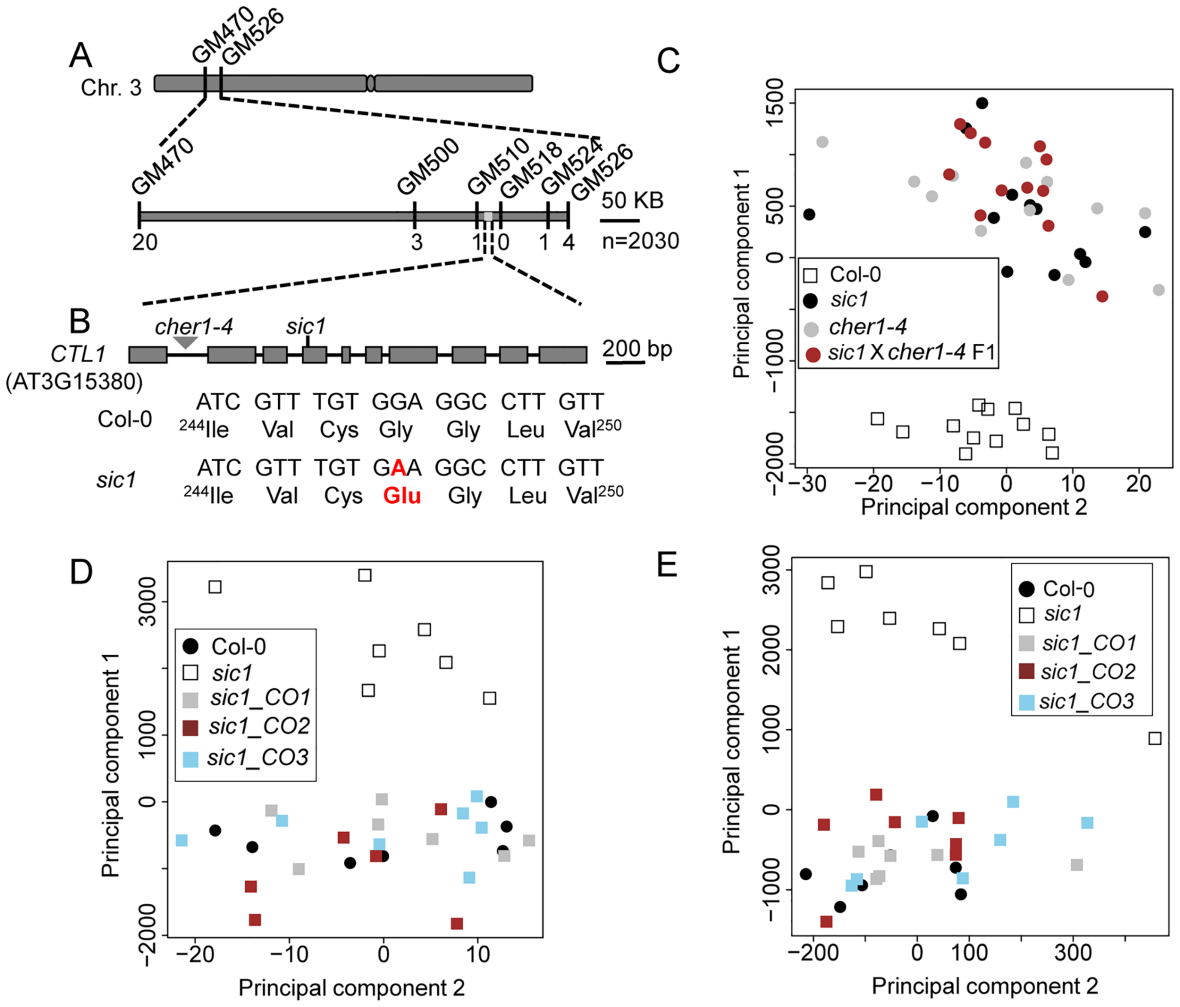
Map-based cloning and complementation of *sic1*. (A) Mapping of *sic1* using 2030 F2 plants narrowed the location of the candidate gene down to a 100-kb region between the markers GM510 and GM524. The numbers under the markers are the number of recombinants between the indicated marker and *sic1*. (B) The gene structure of *CTL1* and the polymorphisms in *sic1* and *cher1-4*. (C) PCA of the leaf ionome of 5-week-old Col-0, *sic1*, and *cher1-4* and the F1 generation of *sic1* × *cher1-4* plants grown in artificial soil. PCA was based on the leaf concentrations of Na, Mn, Fe, Zn, and Mo (*n* = 12). (D-E) PCA of the leaf (D) and root (E) ionomes of 5-week-old Col-0 and *sic1*, as well as three independent transgenic complementation lines. PCA was based on the concentrations of Na, Mn, Fe, Zn, and Mo. *sic1*_CO1, *sic1* _CO2, and *sic1* _CO3 are three independent transgenic lines of *sic1* that were established with wild-type *pSIC1*::*SIC1-GFP* (*n* = 7). All raw data used for creating this figure can be accessed at S1 Data. CTL1, choline transporter-like 1; Col-0, Columbia-0; Cys, cysteine; Fe, iron; Glu, glutamic acid; Gly, glycine; GM, genomic marker; Ile, isoleucine; Leu, leucine; Mn, manganese; Mo, molybdenum; PCA, principle component analysis; *sic1*, significant ionome changes 1; Val, valine; Zn, zinc.

To identify the causal gene of *sic1*, we constructed an F2 mapping population derived from a cross between *sic1* and Landsberg erecta-0 (Ler-0). The ionomic and developmental phenotypes of the F2 individuals co-segregated with wild-type and mutant individuals at a segregation ratio of 3:1 (X^2^ = 0.56 < X^2^_0.05,1_ = 3.84), demonstrating that the ionomic and developmental phenotypes of *sic1* were controlled by the same single recessive locus. Based on the analysis of 2,030 F2 individuals, we mapped the casual locus to a 100-kb region on chromosome 3 (Fig 2A). We sequenced the entire candidate region and identified a single G-to-A mutation on the fourth exon of gene At3g15380. This mutation causes a predicted ^247^Glycine-to-^247^Glutamate substitution in a conserved domain of this protein (Fig 2B and S3 Fig), which suggests that At3g15380 is a good candidate for *SIC1*. To confirm the above finding, we isolated a homozygous transfer DNA (T-DNA) insertion mutant known as *cher1-4* [14], which featured a T-DNA sequence that had been inserted into the first intron of At3g15380 (Fig 2B, S4 Fig). The T-DNA insertion mutant *cher1-4* displayed ionomic and growth phenotypes indistinguishable from those of *sic1* (Fig 2C, S5 Fig and S5 Table). In addition, the F1 progenies of a cross between *sic1* and *cher1-4* displayed phenotypes identical to those of *sic1* and *sic1-2* (Fig 2C, S5 Fig and S5 Table), indicating that *sic1* and *cher1-4* are two alleles of the same locus. In addition, we introduced a functional *pSIC1*::*SIC1-GFP* construct into *sic1* and found that this construct could fully complement both the ionomic and visible phenotypes of *sic1* (Fig 2D, 2E, S5 Fig and S6 and S7 Tables). These data indicated that the mutation identified in At3g15380 is responsible for both the ionomic and the growth defects of *sic1*.

### *SIC1* encodes a choline transporter and is required for PD development

**Fig 3:**
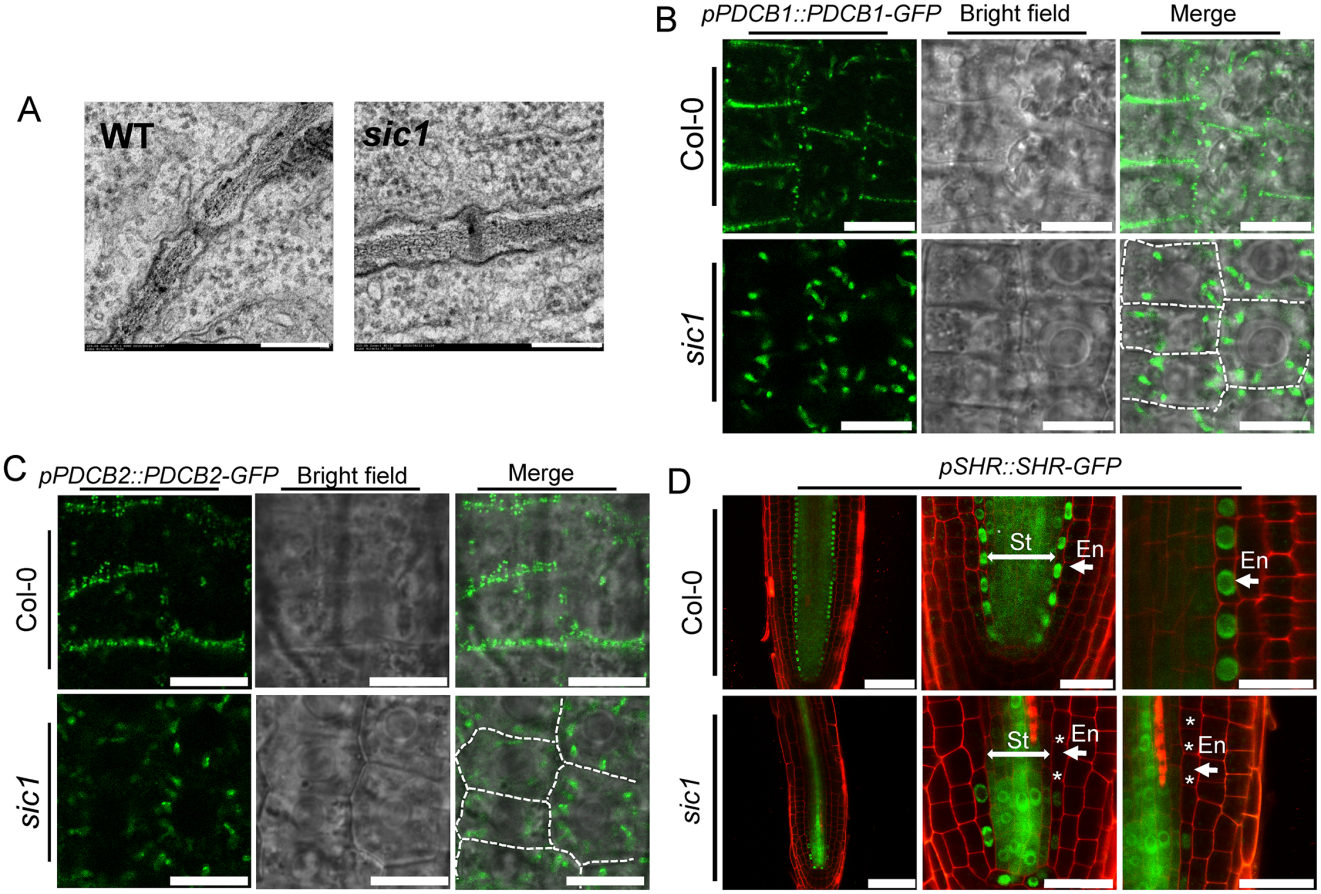
*CTL1* is essential for PD development and regulates intercellular communication. (A) Transmission electron microscopic observations of PDs in the root cortex cells of Col-0 and *sic1*. The scale bars represent 200 nm. (B-C) Subcellular localization of PDCB1 (B) and PDCB2 (C) in Col-0 and *sic1* revealed by *pPDCB1/2*::*PDCB1/2-GFP* expression. The white dashed lines indicate the cell wall. The scale bar represents 10 μm. Five independent transgenic lines of each genotype were observed in this experiment. (D) SHR movement from the St to the En in Col-0 and *sic1*. The green channel shows the SHR-GFP signal, and the magenta channel shows the PI staining signal. The left panel shows the overview of SHR-GFP in Col-0 and *sic1*. The middle panel shows enlarged views of SHR-GFP in quiescent center region of Col-0 and *sic1*, and the right panel shows the absence of SHR-GFP in the endodermal cells of *sic1*. The scale bars are 75 μm for the left panel and 25 μm for middle and right panels. The asterisks represent the endodermal cells lacking a SHR-GFP signal in *sic1*. Six to 10 independent transgenic lines of each genotype were observed in this experiment. CTL1, choline transporter-like 1; Col-0, Columbia-0; En, endodermis; GFP, green fluorescent protein; PD, plasmodesmata; PDCB, PD callose-binding protein; PI, phosphatidylinositol; SHR, shoot root; *sic1*, significant ionome changes 1; St, stele, WT, wild-type.

Sequence analysis showed that *sic1* is a new allele of the recently identified *CTL1* gene, which encodes a choline transporter localized at the TGN and nascent cell plates [14], and the mutation occurs at the second transmembrane domain of the protein (S3 Fig). The *SIC1* gene was therefore renamed *CTL1*. *CTL1* is expressed in both root and shoot, as revealed by quantitative real-time PCR (qRT-PCR) and the green fluorescent protein (GFP) signals in *pCTL1*::*CTL1-GFP* transgenic plants (S6 Fig). In root, CTL1-GFP was observed in all cell types of the root tip, whereas it is mainly distributed in the stele of the maturation zone (S6C Fig). It has been reported that *CTL1* is required for sieve plate and sieve pore formation [14]. As the sieve pore is a special type of PD that plays important roles in ion transportation, we hypothesized that the disorder of ion homeostasis characteristic of *sic1* may be caused by defects in PD. We examined the PD morphology of root cortex cells, wherein PD plays an important role in ion transportation. Using a transmission electron microscope, we observed that most PDs in *sic1* are blocked or shrunken, whereas those in Columbia-0 (Col-0) exhibit typical morphology (Fig 3A). A similar result was also observed in a recent study [18]. To examine PD function in *sic1* further, we examined the localization of two PD markers that play an important role in PD development [19], namely, PDCB1 and PDCB2, by introducing two constructs, *pPDCB1*::*PDCB1-GFP* and *pPDCB2*::*PDCB2-GFP*, into Col-0 and *sic1*. We analyzed five independent transgenic lines for each genotype and construct, and observed well consistent results. Both PDCB1-GFP and PDCB2-GFP were found to be localized predominantly on the cell wall in a spotty manner in wild-type roots, and only a small amount of intracellular fluorescence was observed (Fig 3B and 3C), findings consistent with those of previous studies [19]. In contrast, in the mutant root, either PDCB1-GFP or PDCB2-GFP was localized mostly, if not entirely, in some intracellular compartments, as only a weak PDCB1-GFP signal was observed on the cell wall (Fig 3B and 3C). The intracellular aggregation of PDCB proteins not only confirmed that PDs are defective in *sic1* but also indicated that PDCB could not localize properly in the mutant.

Given that PDs are essential for mineral element transportation and that Casparian strips block the apoplast pathway, it is plausible that PD defects may result in disorders of ion homeostasis. However, we noticed that the levels of some elements were decreased, whereas those of other elements were increased in *sic1* (Fig 1A and 1C, S1–S3 Tables), indicating that impaired PD-mediated symplastic transportation is not the only cause of the defects in ion homeostasis in *sic1*.

A previous study reported that shrunken PD apertures reduce cell-to-cell communication and disrupt processes such as protein and microRNA (miRNA) intercellular translocation and that such changes systematically disturb organism patterning and cell identities [20,21]. Given that *sic1* mutants display a root cell-patterning disorder phenotype (Fig 1F), we wondered whether the intercellular movement of shoot root (SHR), a key transcription factor that moves from the stele to the endodermis to regulate root development, was affected by the shrunken PDs. To validate this hypothesis, we introduced a *pSHR*::*SHR-GFP* construct into Col-0 and *sic1*, and obtained six to ten independent transgenic lines for each genotype for analysis. Confocal microscopic analysis showed that SHR-GFP can move from the stele to the endodermis correctly in all Col-0 transgenic lines, as previously reported [22]. However, PD-mediated intercellular movement was defective in all *sic1* lines, as SHR-GFP was expressed normally in all stele cells but was hardly visible or even vanished in most endodermal cells (Fig 3D). As SHR plays a central role in the initialization and architecture of the cortex, endodermis, and stele [22–24], the reduced movement of SHR noted above may be an important cause of the disordered root patterning characteristic of *sic1* and may result in the abnormal expression of transporters in the wrong cell types.

### The expression patterns of *heavy metal ATPase 4* (*HMA4*) and *high affinity K*^*+*^ *transporter 1* (*HKT1*) were altered in *sic1*

**Fig 4:**
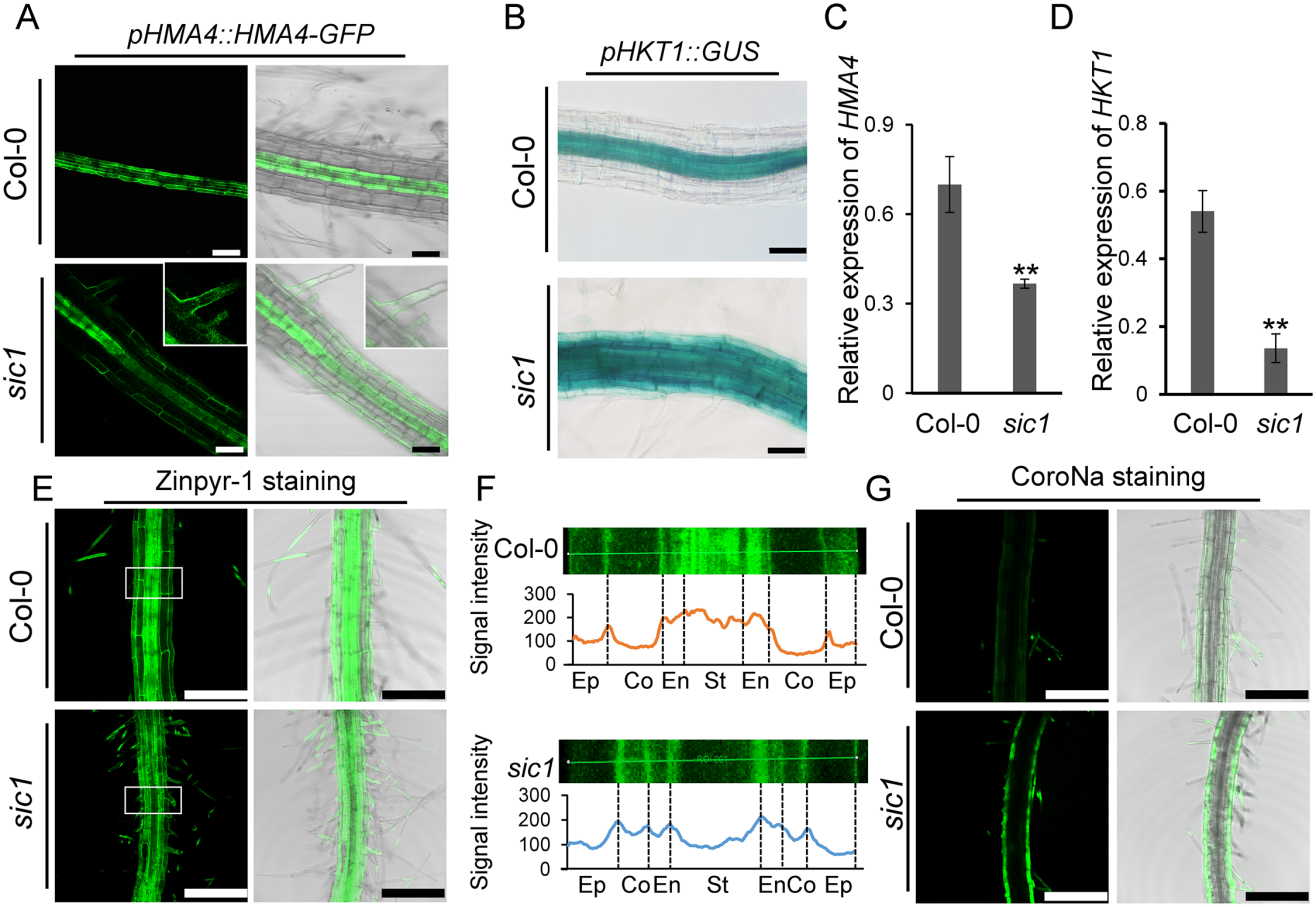
Ectopic expression of *HMA4* and *HKT1* in *sic1* disturbs Zn and Na uptake and transportation. (A-B) The expression patterns of *HMA4* (A) and *HKT1* (B) in Col-0 and *sic1* roots established by *pHMA4*::*HMA4-GFP* (A) and *pHKT1*::*GUS* (B). The insets showed enlarged views of *sic1* root hair. The scale bars represent 50 μm. (C-D) The expression level of *HMA4* (C) and *HKT1* (D) in the roots of Col-0 and *sic1*. The data represents the mean ± SE, *n =* 3. The asterisks above the bar represent a statistically significant difference (*p* < 0.01) calculated using the Student *t* test. (E) The distribution of Zn^2+^ in the roots of Col-0 and *sic1*, as revealed by Zinpyr-1 staining. The scale bars represent 200 μm. (F) The distribution of Zn^2+^ across different cell layers of Col-0 (top) and *sic1* (bottom) roots. The enlarged areas are from the white frames in (E), and the line charts showed the average signal intensity of Zinpyr-1 in different cell layers of Col-0 and *sic1*. The images were captured using the same laser intensity. (G) The distribution of Na^+^ in the roots of Col-0 and *sic1* revealed by CoroNa staining. The scale bar represents 250 μm. The images were captured using the same laser intensity. All raw data used in this figure are included in S1 Data. Co, cortex; Col-0, Columbia-0; CoroNA, CoroNa^™^ Green Sodium Indicator; En, endodermis; Ep, epidermis; GFP, green fluorescent protein; GUS, β-glucuronidase; HMA4, heavy metal ATPase 4; HKT1, high affinity K^+^ transporter 1; Na, sodium; *sic1*, significant ionome changes 1; St, stele; Zn, zinc.

Given that cell-type–specific transporter expression is important for ion homeostasis, we investigated whether the ionomic phenotype of *sic1* was also a result of the ectopic expression of a series of transporters. HMA4 is a Zn^2+^ transporter expressed in the pericycle and xylem parenchyma of roots and is responsible for transporting Zn^2+^ from the symplast to the xylem [25]. As *sic1* exhibits defective Zn^2+^ transport, we assessed the expression patterns of *HMA4* in *sic1* and Col-0. We expressed *pHMA4*::*HMA4-GFP* in *sic1* and introduced the construct into Col-0 by crossing. As previously reported [25,26], HMA4-GFP was clearly present in the pericycle and xylem parenchyma of Col-0 (Fig 4A). However, HMA4-GFP was also found to be ectopically expressed in the *sic1* epidermis, as well as the root hairs of the mature region of the root (Fig 4A). This result was further confirmed by the observation of β-glucuronidase (GUS) signals in 5–8 independent transgenic lines of Col-0 and *sic1*-expressing *pHMA4*::*GUS*. The GUS signal was only present in root steles of Col-0 lines, in which it was also observable in epidermis and root hair cells in addition to the steles of *sic1* lines (S9 Fig). Because HMA4 mediates Zn efflux, we surmised that ectopically expressing *HMA4* in the epidermis of *sic1* would reduce Zn uptake in the root. Interestingly, we found that the total expression level of *HMA4*, as determined by qRT-PCR, was downregulated in *sic1* (Fig 4C), suggesting that Zn loading into the xylem may also be affected in this mutant. To confirm this hypothesis, we stained the roots of Col-0 and *sic1* plants with the membrane-permeant Zn^2+^ fluorescent sensor Zinpyr-1, and found that the distribution of Zn was significantly altered in *sic1* roots compared with Col-0 roots. In contrast to Col-0 roots, which exhibited Zn accumulation mainly in the stele, *sic1* roots exhibited Zn accumulation mainly in the cortex and endodermal cells (Fig 4E and 4F), findings fully consistent with those of the experiments in which disturbances in the expression patterns and reductions in the expression levels of *HMA4* in *sic1* mutants were noted.

HKT1 functions as a Na^+^ transporter in xylem parenchymal cells and is responsible for retrieving Na from the xylem transpiration stream [27,28]. We attempted to express *pHKT1*::*HKT1-GFP* in Col-0 and *sic1* to determine whether, similar to *HMA4*, *HKT1* is ectopically expressed in epidermal cells. However, we failed to observe a GFP signal in either Col-0 or *sic1*. Alternatively, we introduced a *pHKT1*::*GUS* construct into Col-0 and *sic1* mutants. The GUS staining results from six independent lines of each genotype showed that *HKT1* is expressed in the stele of Col-0 roots, as previously reported [29], but is expressed in the epidermis and root hair cells of *sic1* (Fig 4B). Consistent with these findings, we found that *HKT1* was down-regulated in *sic1* (Fig 4D). These results indicated that *HKT1* was also expressed ectopically in *sic1*. It has been shown that ectopic expression of *HKT1* results in increased accumulation of Na in both the roots and the shoot, as it mediates Na influx [1]. We therefore assessed the distribution of Na in Col-0 and *sic1* seedlings by staining CoroNa^™^ Green Sodium Indicator (CoroNa), a membrane-permeant Na fluorescent indicator. We found that Na levels are apparently higher in the root epidermal and cortical cells of *sic1* than in those of Col-0 (Fig 4G).

### CTL1 is required for the subcellular localization of ion transporters

**Fig 5:**
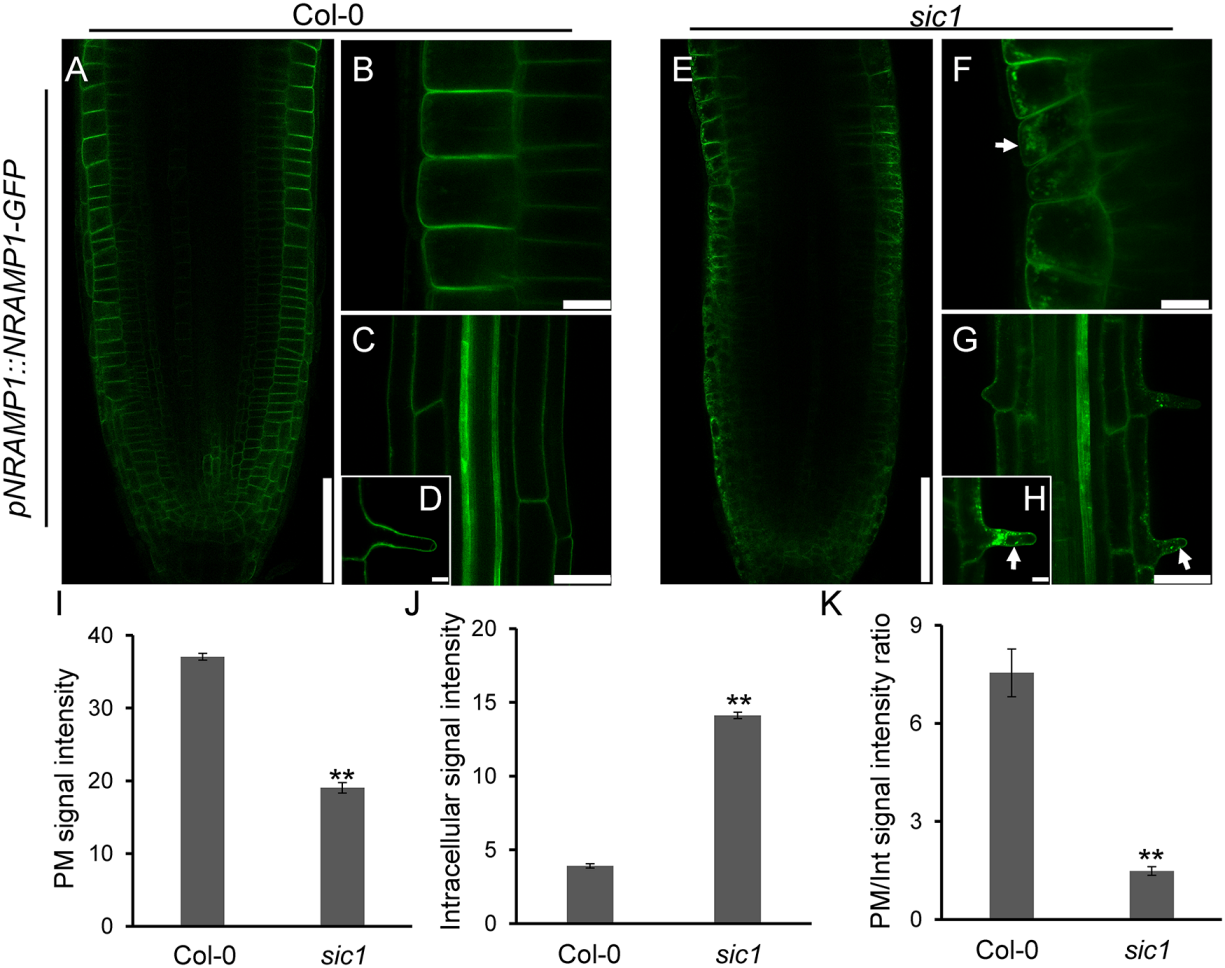
*CTL1* is required for the PM localization of NRAMP1. (A-H) Expression and subcellular localization of NRAMP1 in Col-0 (A-D) and *sic1* (E-H) in root tips (A, B, E, and F) and mature zones (C, D, G, and H). Detailed subcellular localization of NRAMP1 in epidermis cells (B, D) and root hair cells (F, H). The scale bars represent 75 μm in (A, E), 10 μm in (B and F), 50 μm in (C and G), and 10 μm in (D and H). (I-K) Statistical analysis of PM (I) and intracellular (J) NRAMP1-GFP signal and the ratio of PM to intracellular signal (K) in root epidermal cells of Col-0 and *sic1* transgenic plants. Five 6-day-old seedlings of each genotype were used for the calculation, and the images used in this analysis were captured using the same laser intensity. The data represent the means ± SD; the asterisks above the bars represent a statistically significant difference (** indicates *p* < 0.01) calculated using Student *t* test. The white arrows in the pictures indicate intracellular retention of NRAMP1-GFP in *sic1* mutants. All numerical data used in this figure can be found in S1 Data. Col-0, Columbia-0; CTL1, choline transporter-like 1; GFP, green fluorescent protein; NRAMP1, natural resistance-associated macrophage protein 1; Int, intracellular; PM, plasma membrane; *sic1*, significant ionome changes 1.

Given that *sic1* had low concentrations of most of the divalent cations, including Fe^2+^, Mn^2+^, and Zn^2+^, in either the leaves or the roots, we assessed the expression pattern of natural resistance-associated macrophage protein 1 (NRAMP1), a divalent cation transporter responsible for Fe^2+^ and Mn^2+^ uptake and long-distance transportation [30,31]. We expressed a *pNRAMP1*::*NRAMP1-GFP* construct in Col-0 and introduced the construct into *sic1* by crossing *sic1* with transgenic Col-0 plant. Surprisingly, we did not observe alterations in the expression pattern of *NRAMP1* in *sic1*, as both Col-0 and *sic1* expressed *NRAMP1* in the epidermis, cortex, and xylem (Fig 5A–5D), findings consistent with those of a previous report [30]. However, we found that the NRAMP1-GFP protein in *sic1* was intracellularly retained in the epidermis and root hair cells, whereas in Col-0 it was localized predominantly on the PM (Fig 5B, 5D, 5F and 5H). Statistical analysis showed that the PM NRAMP1-GFP signal intensity of Col-0 was 2-fold higher than that of *sic1* (Fig 5I). In addition, the intensity of the intracellular NRAMP1-GFP signal in *sic1* was 4-fold higher than that in Col-0 (Fig 5J), and that the proportion of PM-localized NRAMP1-GFP was 82% lower in *sic1* than in Col-0 (Fig 5K). As NRAMP1 functions in Mn^2+^ and Fe^2+^ uptake and transportation, defects in NRAMP1 trafficking may explain the reduced Mn and Fe concentrations observed in both leaves and roots of *sic1*.

The recycling of iron transporter IRT was previously reported to be mediated by vesicle trafficking [4]. We failed to examine the subcellular localization of IRT1 in *sic1*. But interestingly, we observed that *IRT1* expression was up-regulated up to 9-fold in *sic1* (S10A Fig). Similar to that, the expression of *Arabidopsis* H ^+^-ATPase 2 (*AHA2*), another key gene for iron uptake, is also extremely upregulated in roots of the *sic1* mutant (S5B Fig). These data might reflect that the recycling of IRT1 probably also requires CTL1, and the up-regulation of these genes is just a feedback of mislocalization of their encoding proteins.

### CTL1 is involved in regulating both endocytosis and protein trafficking

**Fig 6:**
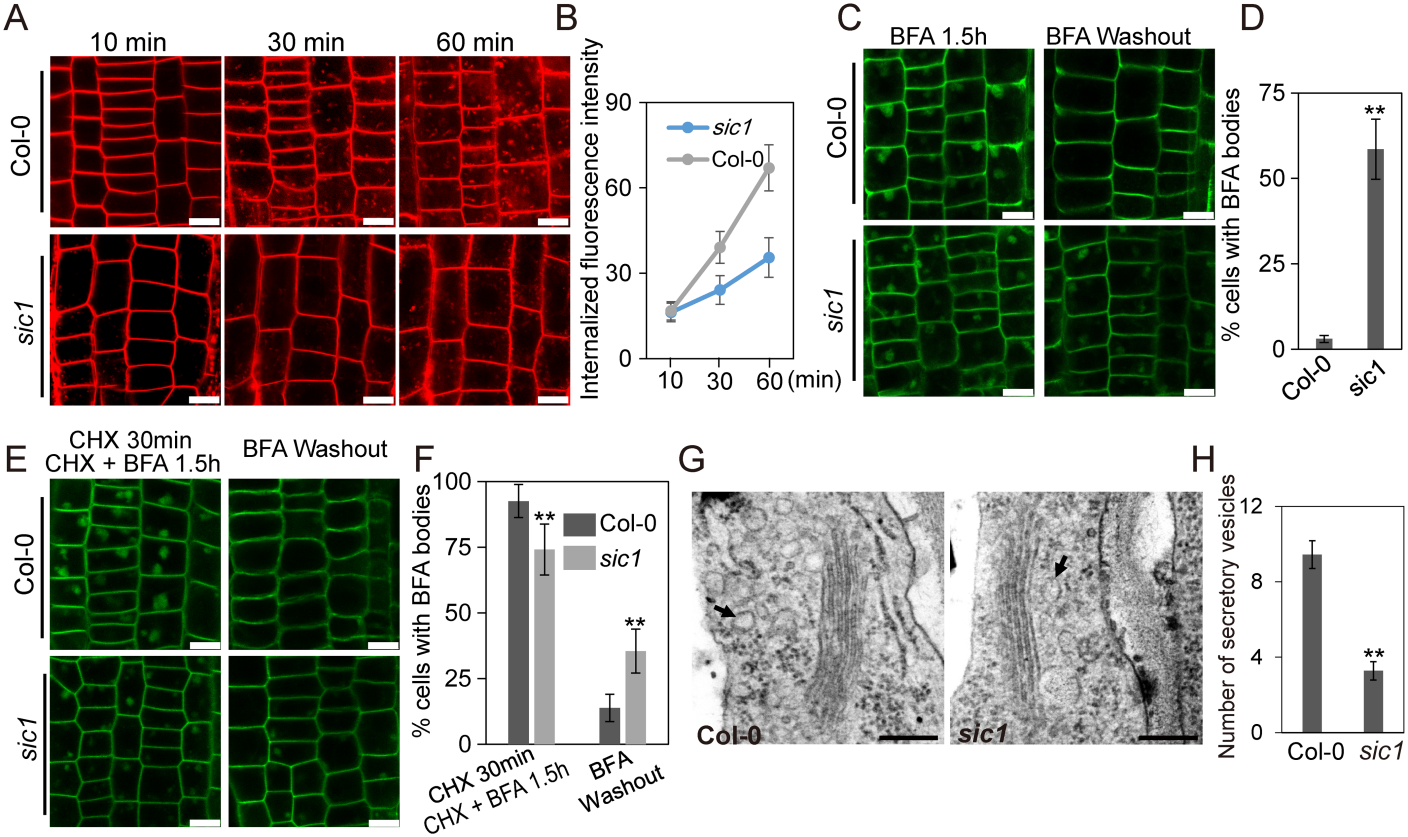
CTL1 mediates endocytosis and secretory trafficking of PM proteins. (A) Internalization of FM4-64 in Col-0 and *sic1*. Images were taken at 10 minutes, 30 minutes, and 60 minutes after FM4-64 staining. The scale bars represent 10 μm. (B) The intracellular signal of FM4-64 in the root cells of Col-0 and *sic1* after FM4-64 staining. The data represent the means ± SD (*n* = 9). The images used in this analysis were captured using the same laser intensity. (C) Recycling of NRAMP1-GFP in the root epidermal cells of Col-0 and *sic1*. The seedlings were treated with 50 μM BFA for 1.5 hours (left panel) and then washed in water for 2 hours (right panel). (D) Statistical analysis of the percentages of root epidermal cells with BFA bodies after 2 hours of washing. The data represent the means ± SD. The asterisks above the bar represent a statistically significant difference (*p* < 0.01) calculated using Student *t* test, *n* = 3. (E) Recycling of NRAMP1-GFP in the root epidermal cells of Col-0 and *sic1* after CHX and BFA treatment. The seedlings were pretreated with 50 μM CHX for 30 minutes and then treated with 50 μM CHX and 50 μM BFA for 1.5 hours and then washed in water for 2 hours. The scale bars represent 10 μm. (F) Statistical analysis of the percentage of root epidermal cells with BFA bodies. The data represent the means ± SD. The asterisks above the bar represent a statistically significant difference (*p* < 0.01) calculated using Student *t* test, *n* = 3. (G) The structure of Golgi apparatus in the root cell of Col-0 and *sic1*. The arrows show the secretory vesicles close to Golgi apparatus. Scale bars represent 200 nm. (H) Quantification of secretory vesicles around Golgi apparatus. The data represent means ± SE. The asterisks above the bar represent a statistically significant difference (*p* < 0.01) calculated using Student *t* test, *n* = 18. The numerical data can be found in S1 Data. BFA, brefeldin A; CHX, cycloheximide; Col-0, Columbia-0; CTL1, choline transporter-like 1; FM4-64, N-(3-Triethylammoniumpropyl)-4-(6-(4-(diethylamino)phenyl)hexatrienyl)pyridinium dibromide; GFP, green fluorescent protein; NRAMP1, natural resistance-associated macrophage protein 1; PM, plasma membrane; *sic1*, significant ionome changes 1.

A previous study reported that CTL1 localizes to the TGN, the hub of vesicle trafficking [14]. Consistent with this finding, we found that CTL1 co-localized with N-(3-Triethylammoniumpropyl)-4-(6-(4-(diethylamino)phenyl)hexatrienyl)pyridinium dibromide (FM4-64), an endocytosis tracer (S7 Fig). This evidence, as well as the observations that PDCB proteins and NRAMP1 are intracellularly aggregated in *sic1*, suggests that CTL1 is probably involved in vesicle trafficking. We stained 6-day-old Col-0 and *sic1* seedlings with FM4-64 to observe endocytosis. Our results showed that FM4-64 internalization was clearly observed after 30 minutes of staining in Col-0 but after 60 minutes of staining in *sic1* (Fig 6A and 6B), indicating that endocytosis was strongly repressed in *sic1*.

Recently, it was reported that the localization of NRAMP1 to the PM was mediated by vesicle trafficking [32]. Considering the intracellular aggregation of NRAMP1 and the suppression of endocytosis in *sic1*, we wondered if CTL1 is involved in regulating the recycling of NRAMP1. To examine this, we treated the transgenic lines expressing *pNRAMP1*::*NRAMP1-GFP* in both Col-0 and *sic1* background with a vesicle trafficking inhibitor brefeldin A (BFA) [33]. BFA inhibits the function of ADP-ribosylation factor GTPases (ARF GTPases) by interacting with their associated guanine nucleotide exchange factors (GEFs) and thereby results in membranous aggregates known as BFA compartments [33]. With BFA treatment for 1.5 hours, the NRAMP1-GFP aggregation in BFA compartments was observed in the epidermal cells of both Col-0 and *sic1* (Fig 6C), supporting that subcellular localization of NRAPM1 is mediated by vesicle trafficking. However, after 2 hours BFA washing out, NRAMP1-GFP aggregation in the BFA compartments only remained in 3.0% of Col-0 epidermal cells but that number in *sic1* is 58.3% (Fig 6C and 6D), suggesting that the recycling of NRAMP1-GFP from endosomes back to PM is altered in *sic1*.

In root, BFA primarily inhibits post-Golgi traffic rather than endoplasmic reticulum (ER) to Golgi traffic due to that this process heavily relies on GNOM-like 1(GNL1), which is BFA resistant [34]. It thus might be contentious that the aggregation of NRAMP1-GFP caused by BFA treatment is from recycling or neosynthesis. To address this question, we first treated the roots of Col-0 and *sic1* expressing *pNRAMP1*::*NRAMP1* with a protein synthesis inhibitor cycloheximide (CHX; 50 μM) for 30 minutes followed by co-treatment with BFA (50 μM) and CHX (50 μM) for 90 minutes. We found that the intracellular BFA bodies in *sic1* were significantly reduced either in amount or in size compared with that in Col-0 after such treatments (Fig 6E and 6F). This observation established that the NRAMP1 recycling is impaired in *sic1* and further confirmed that CTL1 is required for endocytosis. In addition, when the plants were pretreated with CHX (50 μM) for 30 minutes and co-treated with BFA (50 μM) and CHX (50 μM) for 90 minutes followed by 2-hour washing out with water, only 13.9% of BFA bodies remain in Col-0 cells, whereas 35.5% of BFA bodies still remain in *sic1* (Fig 6E and 6F). This data further revealed that recycling of NRAMP1 between endosomes and PM requires CTL1, independently of the de novo delivery.

To further examine the role of CTL1 in vesicle trafficking, we examined recycling of PIN-formed 1(PIN1), an auxin efflux carrier of which polar PM localization is mediated by vesicle trafficking [35]. We pretreated Col-0 and *sic1* plants expressing *pPIN1*:*PIN1-GFP* with CHX for 30 minutes and then co-treated them with CHX and BFA for 90 minutes, and observed BFA bodies in both genotypes—which is consistent with previous studies [36]. Then we washed out the BFA with water to see the protein trafficking of PIN1. After this process, only 14.7% of the BFA bodies still existed in Col-0 cells, whereas 34.8% of the BFA bodies were presented in *sic1* cells (S11 Fig). This result further demonstrated that CTL1 is required for vesicle trafficking and recycling of PM proteins.

As CTL1 localizes on TGN and is involved in vesicle trafficking, we wondered if the intracellularly aggregated NRAMP1 and PDCB proteins in *sic1* are in TGN/early endosomes (EEs). To figure this out, we stained the transgenic line of *sic1* expressing *pNRAMP1*::*NRAMP1-GFP* with FM4-64 for 1 hour, which stains the TGN/EE given 1 hour staining for *sic1* is equivalent to 30 minutes staining for wild type. As expected, we found that the NRAMP1-GFP compartments were indeed co-localized with FM4-64 (S8A Fig), suggesting the aggregation of NRAMP1 is in TGN/EE. To further confirm this, we co-expressed NRAMP1-GFP and the TGN marker VHA-a1-mCherry in *sic1* driven by promoters of *NRAMP1* and *35S*, respectively. Consistently, we observed that the intracellular NRAMP1 compartments were co-localized with the VHA-a1-mCherry, further confirming that NRAMP1 is retained in TGN of *sic1* (S8D Fig). These data suggest that CTL1 is involved in recycling of some cargos from endosomes back to PM. Consistently, when we examined the Golgi apparatus in *sic1* by using a transmission electron microscope, we observed that the numbers of secretory vesicles, a major component of TGN [37], are significantly reduced in *sic1* compared to that in Col-0 (Fig 6G and 6H), suggesting that the TGN structure or its volume might be altered in *sic*. However, when we studied the nature of the intracellular aggregation of PDCBs, we found that they are not the same case as NRAMP1. After staining the transgenic lines of *sic1* expressing PDCB1 with FM4-64, we found the compartments are not co-localized with FM4-64 (S8B and S8C Fig), suggesting that CTL1 might be involved in not only vesicle trafficking-mediated PM protein secretion pathway but also some other unknown secretion pathways.

### Choline homeostasis is crucial for vesicle trafficking

**Fig 7:**
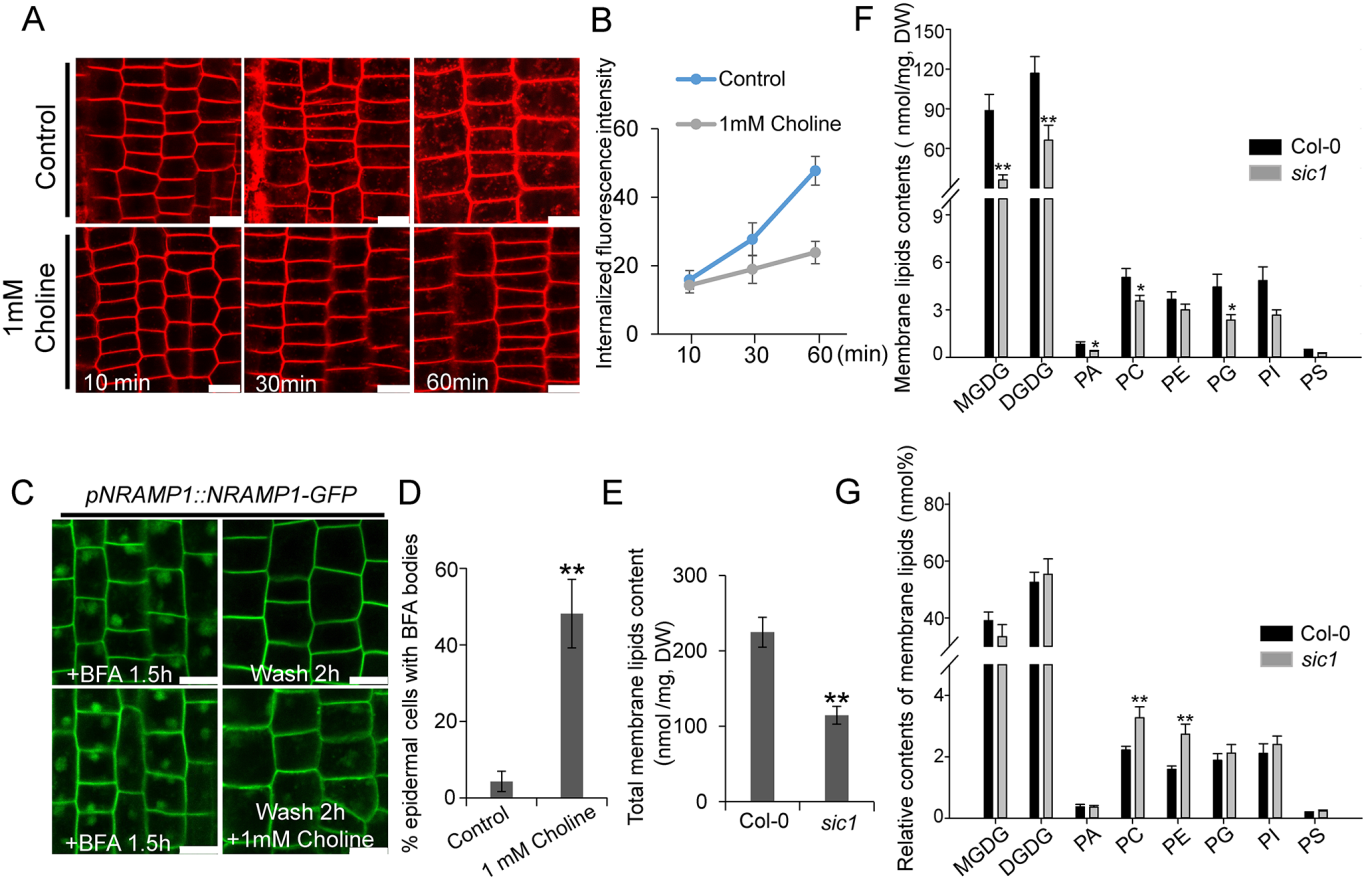
Homeostasis of choline is required for vesicle trafficking. (A) Internalization of FM4-64 in Col-0 root epidermal cells treated with or without 1 mM choline. Images were taken at 10 minutes, 30 minutes, and 60 minutes after FM4-64 staining. The scale bars represent 10 μm. (B) The intracellular FM4-64 signal of Col-0 root epidermal cells treated with or without 1 mM choline. The data represent the means ± SD (*n* = 9 roots). The images used in this analysis were captured using the same laser intensity. (C) Effect of choline on NRAMP1-GFP recycling in the root epidermal cells of Col-0 transgenic plants. The scale bars represent 10 μm. (D) Statistical analysis of the proportions of root epidermal cells with BFA bodies after 2 hours of washing with or without 1 mM choline following BFA treatment. The data represent the means ± SD. The asterisks above the bar represent a statistically significant difference (*p* < 0.01) calculated using Student *t* test (*n* = 8–9 roots). (E) The total membrane lipid contents in Col-0 and *sic1*. The data represent the means ± SE. The asterisks above the bar represent a statistically significant difference (*p* < 0.01) calculated using Student *t* test (*n* = 9 samples). (F) The contents of different membrane lipids in Col-0 and *sic1*. The data represent the means ± SE. Asterisks above the bar represent statistically significant differences (* represents *p* < 0.05, ** represents *p* < 0.01) calculated using Student *t* test (*n* = 9 samples). (G) The relative contents (nmol %) of membrane lipids in Col-0 and *sic1* mutants. The data represent the means ± SE. Asterisks above the bar represent a statistically significant difference (*p* < 0.01) calculated using Student *t* test (*n* = 9–10 samples). The numerical values used in this figure can be accessed at S1 Data. BFA, brefeldin A; Col-0, Columbia-0; DGDG, digalactosyldiacylglycerol; DW, dry weight; FM4-64, N-(3-Triethylammoniumpropyl)-4-(6-(4-(diethylamino)phenyl)hexatrienyl)pyridinium dibromide; GFP, green fluorescent protein; MGDG, monogalactosyldiacylglycerol; NRAMP1, natural resistance-associated macrophage protein 1; PA, phosphatidic acid; PC, phosphatidylcholine; PE, phosphatidylethanolamine; PG, phosphatidylglycerol; PI, phosphatidylinositol; PS, phosphatidylserine; *sic1*, significant ionome changes 1.

CTL1 is a known choline transporter [14]. Therefore, we investigated whether choline or the transporter itself participates in vesicle trafficking by treating 6-day-old Col-0 seedlings with 1 mM choline for 2 hours and then staining the seedlings with FM4-64 dye to observe endocytosis. Interestingly, we found that endocytosis was significantly suppressed under choline treatment (Fig 7A and 7B), suggesting that choline homeostasis is crucial for endocytosis. To investigate the role of choline in protein recycling further, we treated Col-0 seedlings expressing NRAMP1-GFP with 50 μM BFA. After 1.5 hours, we washed the seedlings with either water (control) or 1 mM choline. After 2 hours, 50% of the BFA bodies were still visible in the seedlings washed with choline, whereas 96% of the BFA bodies had vanished in the seedlings washed with water (Fig 7C and 7D). This result suggested that choline homeostasis is important not only for endocytosis but also for membrane protein recycling. In plant cells, choline serves mainly as a precursor of PC, the most abundant phospholipid component of the PM. We thus measured the absolute contents and relative proportions of eight major membrane lipids, including monogalactosyldiacylglycerols (MGDGs), digalactosyldiacylglycerols (DGDGs), PA, PC, phosphatidylethanolamines (PEs), phosphatidylglycerols (PGs), PI, and phosphatidylserines (PS). We found that the absolute contents of all these lipids in *sic1* plants decreased at different levels (Fig 7E and 7F), indicating that the generation or steady status of endomembrane system is impaired in *sic1*. This result is consistent with the role of CTL1 in vesicle trafficking, as vesicle trafficking is required for membrane generation and communication [38].

**Fig 8:**
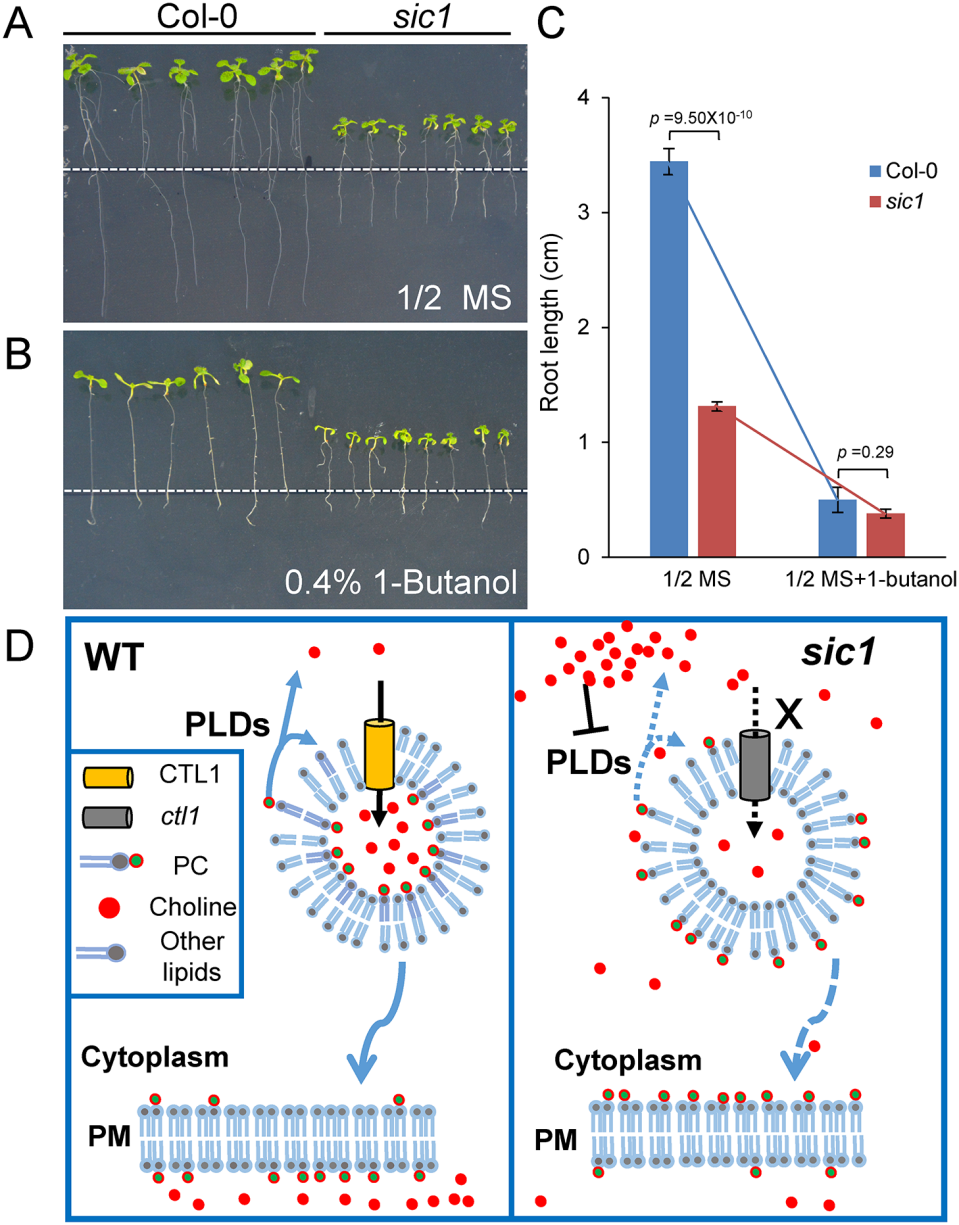
A possible mechanism of *CTL1* in regulation of vesicle trafficking. (A, B) The seedlings of Col-0 and *sic1* grown on 1/2 MS medium (A) and 1/2 MS medium supplemented with 0.4% 1-butanol (B) for five days. The white dashed lines represent the root tip position when seedlings were transplanted. (C) The statistics analysis of the root growth of Col-0 and *sic1* on 1/2 MS with or without 1-butanol treatment. Data represent means ± SE, *n* = 6–7 for each genotype. Asterisks above the bar represent a statistically significant difference (*p* < 0.01) calculated using Student *t* test. (D) A hypothesized model of the mechanism of CTL1 involved in vesicle trafficking. The raw data for this figure can be found in S1 Data. Col-0, Columbia-0; CTL1, choline transporter-like 1; MS, Murashige and Skoog; PC, phosphatidylcholine; PLD phospholipase D; PM, plasma membrane; *sic1*, *significant ionome changes 1*; WT, wild-type.

Interestingly, though the absolute PC and PE contents in *sic1* were decreased, the proportions of PC and PE relative to the total membrane lipids were significantly increased in the mutant, with PC levels increasing by 47% and PE levels increasing by 71% (Fig 7G). This is to say, the compositions of PC and PE in the membrane system of *sic1* increase though they decrease relative to the whole plant. As both PC and PE are substrates of PLDs that cleave PC to form choline, this result may indicate that *CTL1* mutations inhibit PLD activation. To confirm this, we examined the responses of the *sic1* mutant and Col-0 to PLD-specific inhibitor 1-butanol. As expected, *sic1* is more resistant to 1-butanol than Col-0, as the *sic1* root is significantly shorter than Col-0 root when grown on 1/2 Murashige and Skoog (MS) plate, meanwhile there is no significant difference between the roots of the 2 genotypes when treated with 1-butanol (Fig 8A–8C). This result suggested that the PLD activity is inhibited by the mutation of *CTL1*, or in other words, PLD is a downstream component of CTL1. PLDs have been reported to positively regulate vesicle trafficking. We therefore proposed that CTL1 regulates vesicle trafficking through the effects of choline on PLD activity or membrane PC and PE content (Fig 8D).

## Discussion

In this study, we employed a forward genetic-based ionomics approach [16] to determine that the choline transporter CTL1 is a new component of the vesicle trafficking machinery. Interestingly, we found that CTL1 is highly conserved among different species (S3 Fig), indicating that CTL1 plays a crucial and fundamental role in all organisms. Transmembrane domain analysis showed that CTL1 has ten transmembrane helices (S3 Fig, TMHMM Server v. 2.0). And the mutation site lies in the second transmembrane helix, where the mutated glycine is conserved in plants (S3 Fig). Coincidently, a recent study identified the same mutation in CTL1. According to that report, such a mutation results in abnormal subcellular localization of CTL1, which probably explains why this mutation leads to loss of function of *CTL1* [18]. Loss of function of *CTL1* in the root significantly disrupted the leaf homeostasis of a series of mineral nutrients, including Na, Mn, Fe, Zn, and Mo, suggesting that *CTL1* has a systemic impact on ion transportation (Fig 1A–1C). CTL1 was previously shown to be involved in the development of sieve pores, which are a special type of PD [14]. As PDs play an important role in ion transport in the root, it is plausible that the disruption of ion homeostasis characteristic of *sic1* may result from defects in PD. We found that PD development in *sic1* is severely impaired, probably due to PDCB protein mislocalization or some other reasons (Fig 3B and 3C). However, we realized that the direct effects of PDs on ion transport are not specific, as different elements of *sic1* were differentially affected by PD defects. For example, Na^+^ concentrations were increased 3-fold, while Mn^2+^ concentrations were decreased by 77% in *sic1* compared with Col-0 (Fig 1A, 1B and 1C), suggesting that the ionome disorders characteristic of *sic1* may be the ultimate result of several phenomena that exerted direct and indirect effects on ion transport.

Abnormal PDs not only affect ion transport but also block the cell-to-cell communication required for root cell identity, patterning, and development (23). *SHR* is a key transcription factor that initiates root patterning, and its movement from the stele to the endodermis via PDs is essential for its function. We established that SHR cell-to-cell communication is severely blocked in most *sic1* endodermal cells (Fig 3D), leading to abnormal root patterning (Fig 1F). The cell-type specific expression of transporters is generally orchestrated by cell identity and organization, which are driven by certain common transcription factors. Thus, it is reasonable to predict that the expression of some transporters may be altered in *sic1* because of defects in SHR or other mobile transcription factors. We found that both *HMA4* and *HKT1* were ectopically expressed in the epidermis of *sic1* (Fig 4A, 4B and S9 Fig). As a consequence, Zn^2+^ and Na^+^ transport was also disordered (Fig 4E–4G) and may have been an indirect cause of the ionomic phenotype of *sic1*.

Interestingly, not all the transporter genes were misexpressed in the *sic1* mutant, suggesting that the impact of CTL1 on transcription is gene-specific. We noticed that both *HKT1* and *HMA4* are stele-specific genes in Col-0, and their ectopic expression is in epidermal cells of *sic1*, whereas *NRAMP1* is originally expressed in the epidermis. One possibility could be that the expression of the transporter genes like *HKT1* and *HMA4* are inhibited in epidermal cells because of some factors requiring CTL1. In such a case, loss of function of *CTL1* thus could result in disinhibition of *HKT1* and *HMA4* in the epidermis, meanwhile *NRAMP1* is not affected. Consistent with this hypothesis, the establishment of the cell identity of the epidermis also requires factors that move through PDs, such as CAPRICE [39]. In the future, it would be interesting to address whether expression of *HKT1* and *HMA4* are related to these factors and if these transcription factors require CTL1. However, we also noticed that the expression patterns of *HKT1* and *HMA4* are somewhat overlapped with *CTL1* in the mature zone of Col-0, but it is hard to judge if the expression pattern of *CTL1* is related to its gene-specific effect on transcriptional regulation or if that’s just a coincidence.

Although the expression pattern of *NRAMP1* was not changed, we observed that its trafficking to the PM was severely affected in *sic1*. As *NRAMP1* is responsible for Mn and Fe uptake and long-distance transportation, defective NRAMP1 trafficking is certainly at least partially responsible for the low Mn and Fe phenotypes characteristic of *sic1* shoots.

CTL1 localizes to the TGN (S7 Fig) [14], and *CTL1* mutations affect the subcellular localization of various PM proteins, including NRAMP1 and PDCB proteins. These data inspired us to assess whether *CTL1* is involved in regulating vesicle trafficking. Our studies of the endocytosis tracer FM4-64 and NRAMP1 and PIN1 trafficking confirmed our hypothesis. Supporting this conclusion, the numbers of secretory vesicles are much less in *sic1* than in Col-0 (Fig 6G and 6H), which indicates that the TGN structure or volume is changed by the mutation of *CTL1*. The localization of NRAMP1 to the PM was recently reported to be mediated by vesicle trafficking [32]. Our results confirmed this finding and established that this process involves CTL1. In contrast, little is known regarding how PDCB proteins are specifically directed to PDs, which are special PM sites. PDCBs were previously found to be localized to PD domains, where callose deposit and play important roles in PD formation [19]. In this study, we found for the first time that the localization of PDCBs on PDs requires CTL1, which was initially identified as a regulator of the sieve plate and sieve pore and xylem development [14]. As a sieve pore is a special PD in the phloem, the sieve pore phenotype of *ctl1* may also be a result of defects in the subcellular localization of PDCBs or their partner(s).

The *sic1* mutant exhibits a comprehensive ionomic defect, which may indicate that CTL1-mediated vesicle trafficking affects the recycling of large numbers of PM proteins, including various ion transporters. For example, the subcellular localization of the iron transporter IRT1, which is essential for iron uptake of *A*. *thaliana*, was previously found to be regulated by endosomal cycling. Although we did not get direct evidence about the IRT1 localization, we found that the expression of *IRT1*, as well as *AHA2*, was dramatically up-regulated in *sic1* even at Fe sufficient condition (S10 Fig). This elevated IRT1and *AHA2* might reflect that their subcellular localization is also regulated by *CTL1*.

In animals, choline is essential for neuron signaling and choline transporter plays important roles in choline homeostasis [12,13,40]. The vesicle-localized choline transporter was believed to function in secretion of acetylcholine, but it remains unknown if it plays some other roles. It was well-documented that phospholipid compositions of the membrane system play important roles in vesicle trafficking. For example, PA, one of the products in the hydrolysis of PC catalyzed by PLD, is enriched at the budding position of PM to form vesicles [41]. The involvement of PLD in vesicle trafficking was therefore believed to be associated with its role in phospholipid homeostasis on membrane system. Interestingly, as the most abundant phospholipid and the substrate of PLD, PC has never been found to play a role in vesicle trafficking. PC is synthesized on ER or Golgi apparatus and delivered to PM through membrane trafficking. Along this membrane trafficking process, PC contents gradually decreased [42]. In addition, the distribution of PC is asymmetric on PM and endosomes, which predominantly lies on the exoplasmic leaflet of PM and the luminal side of the endosomes. Meanwhile, PC is symmetrically distributed on both sides of the ER membrane [42]. It is unclear what is the significance of these phenomena, but it would be attractive if they represent a mechanism for vesicle trafficking. At least, we might hypothesize that the gradual reduction of PC content during vesicle trafficking is mediated by PC hydrolysis on the cytoplasmic side of the vesicle membrane catalyzed by cytoplasmic PLD. The hydrolysis of PC releases hydrosoluble choline into cytoplasm and leaves PA on the cytoplasmic side of the vesicle membrane. The vesicle-localized choline transporter could be important in this process, given that free choline produced by PC hydrolysis in the cytoplasmic side would inhibit PLD activity. Sequestration of choline into vesicles could help to shape asymmetric distribution of PC on vesicle membrane and PM, and thus to improve vesicle trafficking.

CTL1 was characterized as a TGN-localized choline transporter that regulates choline homeostasis in plant cells [14]. Our findings that CTL1 is involved in vesicle trafficking provide a substantial evidence to support above hypothesis. Interestingly, we observed that the proportions of PE and PC were increased in the cellular membrane system of *sic1*. As PE and PC are both substrates of PLDs, the high proportions of PE and PC in *sic1* may suggest that PLD activity was inhibited in *sic1* mutants, which was then supported by our PLD inhibitor experiment. Thus, it is plausible that CTL1 functions in sequestration of choline into TGN/endosomes to maintain high PA/low PC proportion on the cytoplasmic leaflet and high PC proportion on the luminal side of the vesicles, which might be required for vesicle trafficking. Consistent with this hypothesis, we found that high concentrations of choline inhibit vesicle trafficking. Based on these results, we surmised that the involvement of CTL in vesicle trafficking may be explained by the fact that CTL1 functions in compartmentalizing choline in vesicles to create a low-choline cytosol environment for high PLD activity to promote vesicle trafficking.

Though the relative proportions of PC and PE in the membrane system were increased in *sic1* mutants, the absolute contents of them in the whole plant were both reduced. This result is consistent with previous observation that the absolute content of choline and PC were reduced in *cher1-4* [14]. However, we also found that not only PC, but all other major membrane lipids are decreased in *sic1*. This uniform reduction of all membrane lipids suggests that the mutation of *CTL1* leads to a shrink in producing cellular membrane system. Different membrane organelles are connected through vesicle trafficking. The reduced membrane lipids thus might be a result of vesicle trafficking defects of *sic1*. Of course, we could not exclude the possibility that the reduction of total membrane lipids in *sic1* is caused by lipid synthesis problems, if choline homeostasis could affect synthesis of all membrane lipids.

Except for vesicle trafficking process, the PD development also has a close connection with the lipid composition of the PM-lining PD (PD-PM) domain [43]. In a previous study, it was found that the major phospholipids in PD-PM were PE (approximately 45%) and PC (approximately 20%) [43]. As CTL1 regulates PC and PE homeostasis and their delivering to PM, the mislocalization of PDCBs in *sic1* could also be a result of composition changes of phospholipids on PM. Based on this hypothesis, the intracellular retention of PDCBs in *sic1* might not be a result of vesicle trafficking defect. Otherwise, the subcellular localization of PDCBs in PD should be mediated by some unknown secretory pathway, for example, directly via Golgi apparatus, which has been shown to play an essential role in formation of cell plate and PD [44–46].

Overall, both vesicle trafficking and ion homeostasis are fundamental biological processes. Here, we identified a new participant in both vesicle trafficking and ion homeostasis that links these two critical processes. Furthermore, we provided credible evidence of the existence of a direct chain of molecular events linking vesicle trafficking, ion transporter sorting, PD development, cell-to-cell communication, root patterning, and ion homeostasis. However, the detailed molecular mechanisms underlying the involvement of CTL1 in these processes need to be investigated further.

## Materials and methods

### Plant material and growth conditions

The *A*. *thaliana* plants used in this study were the Col-0 accession, and the T-DNA insertion mutant SALK_065853, which was used to generate *sic1-2*, was obtained from the Nottingham Arabidopsis Stock Centre (NASC). To grow seedlings on agar solidified growth medium, we pretreated *A*. *thaliana* seeds with 75% ethanol for 1 minute, surface-sterilized them by immersing them in 10% NaClO for 10 minutes, and then washed them with distilled water at least five times. The surface-sterilized seeds were sowed on medium containing 1/2 MS salt and 1% sucrose solidified with 0.6% phytalgel (Sigma-Aldrich, St. Louis, MO). After 3 days of stratification at 4°C in the dark, the plates were maintained under 16 hour light/8 hour dark cycles at 23°C.

The plants used for leaf elemental analysis as part of the screening of the EMS-mutagenized plants were grown in a climate-controlled room for up to five weeks, as previously described [15]. The plants were bottom-watered twice a week with modified 0.25 × Hoagland’s Type 2 with 1 mL/L Fe-HBED [15].

Mutagenized *A*. *thaliana* seeds were obtained from Lehle Seeds (Round Rock, TX). To screen 1,554 M2 plants from 17 different parental plants, we grew seed packets and analyzed them by inductively coupled plasma mass spectrometry (ICP-MS). Of these plants, 233 were identified as putative mutants based on their leaf elemental profiles and were allowed to self-fertilize. The seeds were collected, and 11 plants per M3 family were re-screened by ICP-MS after growing in soil. Fifty-six mutants with altered leaf ionomes were identified by this second round of screening.

For hydroponic culture, seeds of *A*. *thaliana* (Col-0 and *sic1*) were stratified for three days at 4°C in water, and then put on a pipe cover with a pore in the middle of a 1.5-mL Eppendorf tube containing Hoagland solution in a climate-controlled room for up to two weeks, as previously described [15]. The seedlings with the pipe cover were then transferred to a new container for 3 additional weeks. The medium was refreshed every three days. The leaves and roots of 5-week-old plants were collected for elemental analysis.

### Elemental analysis

*A*. *thaliana* leaf tissue elemental analysis via ICP-MS has been previously described [47]. Briefly, 2 healthy rosette leaves from one 5-week-old plant were cut with a scalpel, and the leaf was held with plastic tweezers. The collected leaves were subsequently rinsed with 18 MΩ water in a 1,000-ml beaker four times to wash off external impurities. The rinsed samples were then placed in glass tubes and pushed to the bottoms of the tubes with a glass rod to ensure that none of them were left on the tube walls. The tubes were then transferred to an oven for 20 hours at 92°C. After cooling, seven to ten samples were weighed on an analytical balance. All the samples, including the blank controls, were then digested with 1 ml of concentrated nitric acid containing indium (In) as an internal standard for 4 hours at 110°C before being diluted with 18 MΩ water in a final volume of 10 ml. Elemental analysis of Li, B, Na, Mg, P, S, K, Ca, Mn, Fe, Co, Ni, Cu, Zn, As, Se, Rb, Sr, Mo, and Cd was performed with an ICP-MS (NexION 350D; PerkinElmer, Waltham, MA) coupled with an Apex desolvation system and an SC-4 DX autosampler (Elemental Scientific Inc., Omaha, NE). All the samples were normalized with a heuristic algorithm using the best measured elements as previously described [15].

### *A*. *thaliana* grafting

Reciprocal grafting was performed as previously described [47]. After the graft unions were established, the grafted plants were examined under a stereoscopic microscope before being transferred into potting mix soil to observe the formation of any adventitious roots from the graft unions or above. Healthy grafted plants without adventitious roots were transferred to potting mix soil and grown in the controlled environment described above. After four weeks, leaf samples were harvested for ionomic analysis. After harvesting, the plants were examined again, and those with adventitious roots or without a clear graft union were excluded from the subsequent analysis of the ionomic data.

### Map-based cloning of *sic1*

PCR-based genotyping was used for mapping of the causing mutation in *sic1*. Using two SSLP markers and a population of 262 F2 plants from the Ler-0 Χ sic1 cross, we were able to map the gene to within a 560-kb genomic region on chromosome 3. Further SSLP markers were developed within this 560-kb mapping interval and used to screen 1,768 further Ler-0 Χ sic1 F2 plants to identify informative recombinants to further narrow the mapping interval to a 100-kb region between SSLP markers of GM510 and GM524. Overlapping fragments of approximately 0.7 to approximately 1 kb each, covering this 80-kb candidate region, were amplified from the genome of *sic1* and sequenced. The sequence of these fragments was alignment with the wild-type sequence using KOD neo plus (TOYOBO, Osaka, Japan). The markers used in identifying informative recombinants were shown in S8 Table.

### Vector construction and transgenic plant development

For the *sic1* complementation test, we constructed a *pSIC1*::*SIC1-GFP* fusion vector. A 4.4-kb genomic DNA fragment including a 1.3-kb gene promoter and a gene body without a TGA stop codon was amplified using the primers SIC1-GFP-L and SIC1-GFP-R, and the GFP fragment was amplified from the *pHAC1*::*HAC1-GFP* vector [47] using the primers GFP-L and GFP-R. Thereafter, the two fragments were fused together by overlapping PCR using the primers SIC1-GFP-L and GFP-R. The fused fragment was inserted into a *pHMS* plant expression vector modified from a *pHB* vector [48] via *Hind*III and *Pst*I restriction sites using a Hieff Clone one-step PCR cloning kit (Yisheng Co. Ltm, Shanghai, China).

To construct the *pHMA4*::*HMA4-GFP* fusion vector, we amplified an approximate 10-kb *HMA4* genomic DNA fragment from Col-0 by PCR using a KOD Hot Start DNA Polymerase (TOYOBO). The fragment included a 4-kb gene promoter and gene body but lacked a TGA stop codon. Amplification was performed using the primer pair HMA4-GFP-L and HMA4-GFP-R. Because of the large size of the *HMA4* genomic DNA fragment, the GFP coding sequence fused with five GGA repeats at the N-terminal, where it served as a linker, was cloned into the *pHMS* vector backbone between the *Pst* I and *Xba* I restriction sites in advance. This modified *pHMS* vector was renamed as *pGHMS*. The amplified *HMA4* fragment was cloned into *pGHMS* using *Hind* III and *Pst*I restriction sites using a Hieff Clone one-step PCR cloning kit (Yisheng Co. Ltm).

For the *pNRAMP1*::*NRAMP1-GFP* construct, we prepared the *NRAMP1* genomic DNA, including the 2.5-kb native promoter, from Col-0 plants by PCR amplification using the primers NRAMP1-GFP-L and NRAMP1-GFP-R. The amplified fragment was cloned into a *pGHMS* vector with *Hind* III and *Pst* I restriction sites using a Hieff Clone one-step PCR cloning kit (Yisheng Co. Ltm).

For the *35S*::*VHA-a1-mCherry* construct, we first modified the *pHB* vector [48] by inserting the mCherry fragment with a five-glycine linker between the *Pst* I and *Xba* I restriction sites, and renamed the modified *pHB* vector as *pHB-mCherry*. We then amplified the CDS region of *VHA-a1* from the Col-0 cDNA using the primers VHA-a1-mCherry-L and VHA-a1-mCherry-R. The amplified CDS fragment was then cloned into *pHB-mCherry* vector with *Hind* III and *Pst* I restriction sites using a Hieff Clone one-step PCR cloning kit (Yisheng Co. Ltm).

To construct the *pHKT1*::GUS vector, we amplified the *HKT1* promoters with the primer pair HKT-GUSL and HKT-GUSR using Col-0 genomic DNA as a template, after which we inserted them into the pCAMBIA1303 vector to drive uidA expression. GUS histochemical staining was performed as previously described [30].

The procedure with which the *pSHR*::*SHR-GFP* and *pPDCB1*::*PDCB1-GFP* and *pHMA4*::*GUS* vectors were constructed has been previously described [19,49].

All the expression vectors were transformed into the *Agrobacterium tumefaciens* strain GV3101 and introduced into Col-0 or *sic1* using the floral dip method [50].

The primers used in the study are shown in S8 Table.

### qRT-PCR

The roots of 3-week-old hydroponically cultured Col-0 and *sic1* plants were used to extract total RNA by using TRNzol A+ RNA Purification reagent (DP421; Tiangen Biotech, Beijing, China). Two micrograms of total RNA were used to synthesize first-strand cDNA with TransScript one-step gDNA removal and cDNA synthesis super mix (AT311-02; TransGen Biotech, Beijing, China). qRT-PCR was performed using SYBR green PCR master mix (TRT-101; TOYOBO) with the first-strand cDNA as a template on a real-time PCR system (CFX thermocycler; Bio-Rad, ‎Hercules, CA). Primers for qRT-PCR were designed using Primer Express software version 3.0 (Applied Biosystems, Foster City, CA). The primers UBCF and UBCR were designed for ubiquitin-conjugating enzyme 21 (At5g25760), which was used as the reference gene. The primer sequences are shown in S8 Table. Expression data analysis was performed as previously described [51].

### Chemical treatment

Before choline treatment, choline chloride was dissolved in water to make a 100-mM choline solution. In the experiment, the choline solution was diluted to a concentration of 1 mM, and the seedlings were treated for the desired time.

For BFA treatment, 6-day-old *Arabidopsis* seedlings were immersed in 50 μM BFA for 1.5 hours, and then the BFA bodies were observed by confocal laser scanning microscopy (Leica TCS SP8). In the washout experiments, the BFA-treated seedlings were incubated in water for 2 hours, after which the BFA bodies were examined by confocal laser scanning microscopy (Leica TCS SP8).

For CHX and BFA treatment, 6-day-old *Arabidopsis* seedlings were pretreated with 50 μM CHX for 30 minutes, followed by being immersed in a solution with 50 μM CHX and 50 μM BFA for 1.5 hours, and then the BFA bodies were observed by confocal laser scanning microscopy (Leica TCS SP8). In the washout experiments, above seedlings were incubated in water for 2 hours, and the BFA bodies were then examined by confocal laser scanning microscopy (Leica TCS SP8).

For 1-butanol treatment, the *Arabidopsis* seeds were grown on 1/2 MS salt medium plate for six days, and the seedlings were then transplanted to the 1/2 MS salt medium plate containing 0.4% 1-butanol and grew for another five days. The root length was measured by Image J.

### CoroNa staining and observations

Seven-day-old Col-0 and *sic1* plants grown on 1/2 MS plates were treated with PBS solution containing 20 mM Zinpyr-1 (Cayman Chemical, Ann Arbor, MI) at room temperature in darkness for 3 hours. Then, the seedlings were washed with PBS solution twice. The stained plants were observed with a confocal laser scanning microscope (Leica TCS SP8) using excitation at 490 nm and emission at 530 nm.

For CoroNa staining, 6-day-old Col-0 and *sic1* plants grown on 1/2 MS plates were treated with 5 μM CoroNa water solution in the dark for 20 minutes. The stained plants were washed in water twice and then observed under a confocal laser scanning microscope (Leica TCS SP8) with an excitation wavelength of 492 nm and an emission wavelength of 516 nm.

### Microscopy observation

Confocal laser scanning microscopy was performed on Leica TCS SP8 and Olympus FluoView FV1000 confocal microscopes. To observe the GFP fusions, we illuminated 5- to 7-day-old plants with an excitation wavelength of 488 nm with an Argon laser, and emission was detected at 505–550 nm. The images used for the comparisons of signal intensity between Col-0 and *sic1* were captured using a confocal microscope with the same settings. For the FM4-64 internalization assay, 5- to 7-day-old Col-0 and *sic1* mutant seedlings grown on solidified growth medium were incubated with 2 μM FM4-64 solution for 5 minutes and then rinsed twice with water. The concentration of the FM4-64 stock solution in DMSO was 4 mM. The FM4-64 in seedlings was visualized at 10 minutes, 30 minutes, and 60 minutes after staining. For propidium iodide staining, 5-day-old seedlings were incubated in a fresh solution of 15 mM (10 mg/ml) PI dissolved in water in the dark for 5 minutes, after which they were rinsed twice in water. The fluorescence excitation and emission wavelengths of FM4-64 and PI were 561 nm and 610–630 nm, respectively. The fluorescence signal intensity was calculated use Leica Application Suite X (version 1.1.0, Leica, Wetzlar, Germany). The PDs were imaged at 80 kV with a Hitachi H-7650 transmission electron microscope.

### Lipid extraction and determination

The extraction and analysis of membrane lipids were performed as described previously [52]. Briefly, the seedlings were quickly immersed in isopropanol preheated to 75°C with 0.01% butylated hydroxytoluene (BHT) for 15 minutes. Then chloroform and water (5:2) were added, and the mixture was incubated at room temperature for 1 hour. The lipid extract was transferred into glass tubes with Teflon-lined screw-caps. Chloroform/methanol (2:1) with 0.01% BHT was added into the extracts and shaken for 30 minutes. This process was repeated until all samples became white. The extracts for each sample were combined and washed with 1 M KCl followed by water washing. The final lipid extractions were evaporated under a gentle flow of N_2_ gas and then stored at −80°C. The membrane lipid analysis was performed by automated electrospray ionization–tandem mass spectrometry as previously described, with the standards including di14:0-PC, di24:1-PC, 13:0-lysoPC, 19:0-lysoPC, di14:0-PE, di24:1-PE, 14:0-lysoPE, 18:0-lysoPE, di14:0-PG, di24:1-PG, 14:0-lysoPG, 18:0-lysoPG, di14:0-PA, di20:0(phytanoyl)-PA, di14:0-PS, di20:0(phytanoyl)-PS, 16:0–18:0-PI, di18:0-PI, 16:0–18:0-MGDG, di18:0-MGDG, 16:0–18:0-DGDG, and 0.71 nmol di18:0-DGDG [53].

## Supporting information

S1 Fig The *sic1* mutant exhibits developmental defects in rosette leaves. (A) The 3-week-old *sic1* mutant grown on artificial soil shows growth retardation compared to Col-0. Scale bar represents 3 cm. (B) The rosette leaf number of Col-0 and sic1. Scale bar represents 2 cm. Col-0, Columbia-0; *sic1*, significant ionome changes 1.
(TIF)

S2 Fig The leaf developmental phenotype of *sic1* is partially driven by root. The images were taken on the 20th day after grafting. Col-0/*sic1*, grafted plants with Col-0 shoot and *sic1* root; *sic1*/Col-0, grafted plants with *sic1* shoot and Col-0 root. Col-0, Columbia-0; NG, non-grafted plants; SG, self-grafted plants; *sic1*, significant ionome changes 1.
(TIF)

S3 Fig CTL1 is highly conserved among different species. Protein sequence alignment of CTL1 among different species shows CTL1 is highly conserved and the mutation site of *sic1* is located in a conserved domain. And the red boxes show the TMHs. The red triangle indicates the mutation site of *sic1*. CTL1, choline transporter-like 1; *sic1*, significant ionome changes 1; TMH, transmembrane helix.
(TIF)

S4 Fig The expression of *CTL1* in *sic1* and *cher1-4*. (A) The insertion site of *cher1-4* and the position of primers used in this experiment. The triangle showed the insertion site of *cher1-4* and the arrows shows the positions of primers used in this experiment (B) RT-PCR analysis of *CTL1* transcripts in Col-0, *sic1*, and *cher1-4*. *UBC* was used as an internal standard for the RT-PCR. Single PCR reactions were performed on RNA from individual plants of each genotype. (C) The qRT-PCR result of *CTL1* in Col-0, *sic1* and *cher1-4*. The data represent the means ± SE; *n* = 3. The raw data can be found in S1 Data. Col-1, Columbia-0; CTL1, choline transporter-like 1; PCR, polymerase chain reaction; qRT-PCR, quantitative real-time polymerase chain reaction; RT-PCR, real-time polymerase chain reaction; *sic1*, significant ionome changes 1.
(TIF)

S5 Fig Genetic and transgenic complementation of developmental phenotype *sic1*. (A) The 6-day-old seedlings of Col-0, *sic1*, *cher1-4*, and complementation lines grown on agar-solidified 1/2 MS medium plate. Scale bar represents 1 cm. (B) Primary root length of Col-0, *sic1*, *cher1-4*, and complementation lines. Letters above bars indicate statistically different groups using a one-way ANOVA, followed by an LSD test at the probability of *p* < 0.05. Data represent means ± SE, *n* = 10 for each genotype. (C) Root patterning of Col-0 and *sic1_*CO plants. Scale bars represent 50 μm. The raw data can be found in S1 Data. Col-0, Columbia-0; LSD, least significant difference; *sic1*, significant ionome changes 1.
(TIF)

S6 Fig The expression pattern of *CTL1* in different tissues of *A*. *thaliana*. (A-D) CTL1-GFP showed the expression pattern in root tip (A), root maturation zone (B and C), leaf pavement cells (D). Three independent transgenic lines were observed and showed the same expression pattern. Green channel represents CTL1-GFP signal and red channel represents propidium iodide signal. Scale bars represent 50 μm for (A) and (D). (E) The relative expression of *CTL1* in the shoot and root of Col-0 plants. Data represent means ± SE, *n* = 3. The raw data can be found in S1 Data. CTL1, choline transporter-like 1; GFP, green fluorescent protein.
(TIF)

S7 Fig CTL1 localizes to PM and endosome membrane vesicles. (A) An enlarged view of CTL1-GFP in the root tip of Col-0 plant. Green channel shows the GFP signal and red channel shows the PI signal. Scale bar represents 10 μm. (B) Subcellular localization of CTL1-GFP after plasmolysis in Col-0 root. Green channel shows the GFP signal. Scale bar represents 50 μm. (C) CTL1 is co-localized with FM4-64 in root cells. Green channel shows the CTL1-GFP signal; red channel shows the FM4-64 signal. The insets showed an enlarged view of epidermal cell. Scale bars represent 5 μm. Col-0, Columbia-0; CTL1, choline transporter-like 1; GFP, green fluorescent protein; FM4-64, N-(3-Triethylammoniumpropyl)-4-(6-(4-(Diethylamino) Phenyl) Hexatrienyl) Pyridinium Dibromide; PM, plasma membrane.
(TIF)

S8 Fig The aggregation of NRAMP1 and PDCB proteins locate in different intracellular organelles. (A) The intracellular aggregation of NRAMP1-GFP is co-localized with FM4-64 signal in *sic1*. (B and C) The intracellular aggregations of PDCB1-GFP (B) and PDCB2-GFP (C) are not co-localized with FM4-64 in *sic1*. The transgenic seedlings of *sic1* background were stained with FM4-64 for 1 h and then observed in confocal microscope. Green channels represent the GFP signal, and red channels represent the FM4-64 signal. The scale bars represent 7.5 μm in (A) and 5 μm in (B and C). (D) The intracellular aggregation of NRAMP1-GFP in *sic1* localized to TGN. The scale bars represent 7.5 μm. Col-0, Columbia-0; CTL1, choline transporter-like 1; GFP, green fluorescent protein; FM4-64, N-(3-Triethylammoniumpropyl)-4-(6-(4-(diethylamino)phenyl)hexatrienyl)pyridinium dibromide; NRAMP1, natural resistance-associated macrophage protein 1; PDCB, plasmodesmata callose-binding protein; *sic1*, significant ionome changes 1.
(TIF)

S9 Fig The expression pattern of *HMA4* in the roots of Col-0 and *sic1*. The insets showed an enlarged view of *sic1* root hair. Scale bars represent 50 μm. Col-0, Columbia-0; HMA4, heavy metal ATPase 4; *sic1*, significant ionome changes 1.
(TIF)

S10 Fig The expression of iron uptake related genes in the roots of Col-0 and *sic1*. (A-B) The expression levels of *IRT1* (A) and *AHA2* (B) in the roots of Col-0 and *sic1*. The data represent the mean ± SE, *n = 3*. The asterisks above the bar represent a statistically significant difference (*p* < 0.01) calculated using Student *t* test. The raw data can be found in S1 Data. *AHA2*, *Arabidopsis* H ^+^-ATPase 2; Col-0, Columbia-0; *IRT1*, iron regulated transporter 1; *sic1*, significant ionome changes 1.
(TIF)

S11 Fig The recycling of PIN1 was affected in *sic1* mutant. (A) The BFA compartments were observed after CHX and BFA treatment in Col-0 and *sic1*. (B) The subcellular localization of PIN1 in Col-0 and *sic1* after BFA washout. Scale bars represent 20 μm. (C) The statistics analysis of percentage of cells with BFA bodies. The data represent the mean ± SE, ten seedlings of three independent transgenic lines were used in this analysis. The asterisks above the bar represent a statistically significant difference (*p* < 0.01) calculated using Student *t* test. The raw data can be found in S1 Data. BFA, brefeldin A; CHX, cycloheximide; Col-0, Columbia-0; PIN1, PIN formed 1; *sic1*, significant ionome changes 1.
(TIF)

S1 Table Leaf ionome of Col-0 and *sic1* grown in artificial soil. Data represents means ± SE, *p*-values were calculated by Student *t* test, *n* = 12. Col-0, Columbia-0; *sic1*, significant ionome changes 1.
(XLSX)

S2 Table Leaf ionome of Col-0 and *sic1* grown in Hoagland’s solution. Data represents means ± SE, *p*-values were calculated by Student *t* test, *n* = 7. Col-0, Columbia-0; *sic1*, significant ionome changes 1.
(XLSX)

S3 Table Root ionome of Col-0 and *sic1* grown in Hoagland’s solution. Data represents means ± SE, *p*-values were calculated by Student *t* test, *n* = 7. Col-0, Columbia-0; *sic1*, significant ionome changes 1.
(XLSX)

S4 Table Leaf ionome of Col-0 and *sic1* reciprocal grafting experiment. Data represents means ± SE. *sic1* /Col-0, grafted plants with *sic1* shoot and Col-0 root; Col-0/*sic1*, grafted plants with Col-0 shoot and *sic1* root. *n* = 7–19. Col-0, Columbia-0; NG, non-grafted plants; SG, self-grafted Col-0; *sic1*, significant ionome changes 1.
(XLSX)

S5 Table Leaf ionome of genetic complementation. Data represents means ± S.E., *n* = 12 for each genotype.
(XLSX)

S6 Table Leaf ionome of *sic1* transgenic complementation. Data represents means ± SE, *sic1* _CO1, *sic1* _CO2 and *sic1* _CO3, three independent transgenic complementation lines of *sic1* with wild-type *pSIC1*::*SIC1-GFP*, *n* = 7 for each genotype. Col-0, Columbia-0; *sic1*, significant ionome changes 1.
(XLSX)

S7 Table Root ionome of *sic1* transgenic complementation. Data represents means ±S.E., *sic1* _CO1, *sic1* _CO2 and *sic1* _CO3, three independent transgenic complementation lines of *sic1* with wildtype *pSIC1*::*SIC1-GFP*, *n* = 7 for each genotype. Col-0, Columbia-0; *sic1*, significant ionome changes 1.
(XLSX)

S8 Table Primers used in this study (from 5ʹ–3ʹ). (XLSX)

S1 Data Data used to generate the figures. (XLSX)

## Abbreviations

AHA2: *Arabidopsis* H +-ATPase 2
ARF GTPase: ADP-ribosylation factor GTPase
AtHKT1: *Arabidopsis thaliana* high affinity K^+^ transporter 1
B: boron
BFA: brefeldin A
BHT: butylated hydroxytoluene
Ca: calcium
Cd: cadmium
CHX: cycloheximide
Co: cobalt
Col-0: Columbia-0
CoroNA: CoroNa^™^ Green Sodium Indicator
CTL1: choline transporter-like 1
Cu: copper
DGDG: digalactosyldiacylglycerol
EE: early endosome
EMS: ethyl methanesulfonate
ER: endoplasmic reticulum
Fe: iron
FM4-64: N-(3-Triethylammoniumpropyl)-4-(6-(4-(diethylamino)phenyl)hexatrienyl)pyridinium dibromide
FYVE1: FYVE domain protein required for endosomal sorting 1
GEF: guanine nucleotide exchange factor
GFP: green fluorescent protein
GNL1: GNOM-like 1
GUS: β-glucuronidase
HKT1: high affinity K^+^ transporter 1
HMA4: heavy metal ATPase 4
ICP-MS: inductively coupled plasma mass spectrometry
IDF1 RING: IRT1 degradation factor1 RING-type
In: indium
IRT1: iron regulated transporter 1
K: potassium
Ler-0: Landsberg erecta-0
Li: lithium
MGDG: monogalactosyldiacylglycerols
miRNA: microRNA
Mn: manganese
Mo: molybdenum
MS: Murashige and Skoog
Na: sodium
NASC: Nottingham Arabidopsis Stock Centre
Ni: nickel
NRAMP1: natural resistance-associated macrophage protein 1
P: phosphorus
PA: phosphatidic acid
PCA: principle component analysis
PCs: phosphatidylcholine
PD: plasmodesmata
PDCB: plasmodesmata callose-binding protein
PD-PM: plasma membrane-lining plasmodesmata
PE: phosphatidylethanolamine
PG: phosphatidylglycerol
PI: phosphatidylinositol
PI3P: phosphatidylinositol-3-phosphate
PIN1: PIN-formed 1
PLD: phospholipase D
PM: plasma membrane
PS: phosphatidylserine
qRT-PCR: quantitative real-time PCR
S: sulfur
SHR: shoot root
*sic1*: significant ionome changes 1
T-DNA: transfer DNA
TGN: trans-Golgi network
Zn: zinc
