# Supplementary figures and images for: A new vesicle trafficking regulator CTL1 plays a crucial role in ion homeostasis
Source: PLoS Biol. 2017 Dec 28;15(12):e2002978. doi: 10.1371/journal.pbio.2002978 (PMC5746208; doi:10.1371/journal.pbio.2002978)

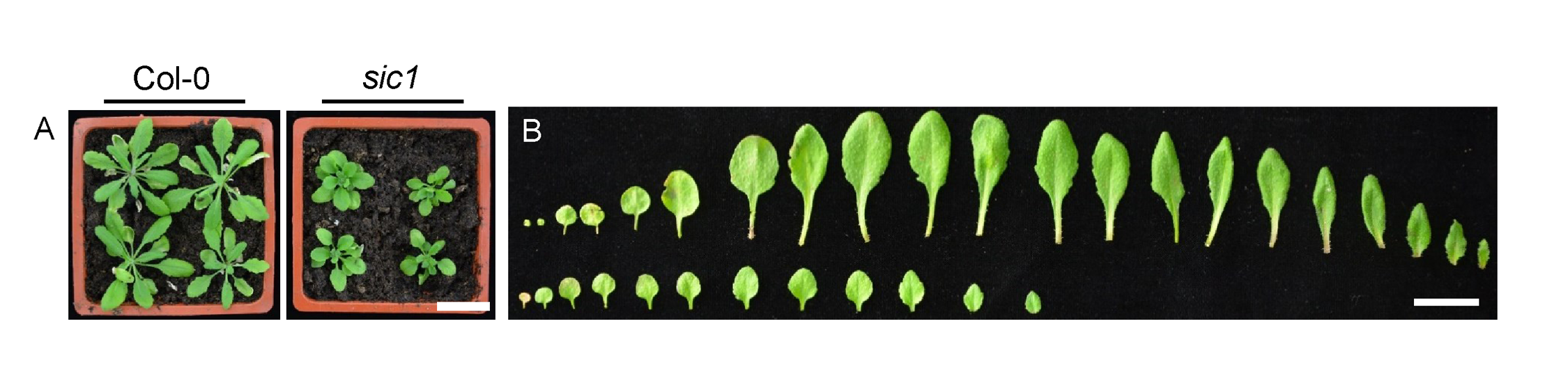

Supplement: S1 Fig — (A) The 3-week-old sic1 mutant grown on artificial soil shows growth retardation compared to Col-0. Scale bar represents 3 cm. (B) The rosette leaf number of Col-0 and sic1. Scale bar represents 2 cm. Col-0, Columbia-0; sic1, significant ionome changes 1. (TIF) [file pbio.2002978.s001.tif]

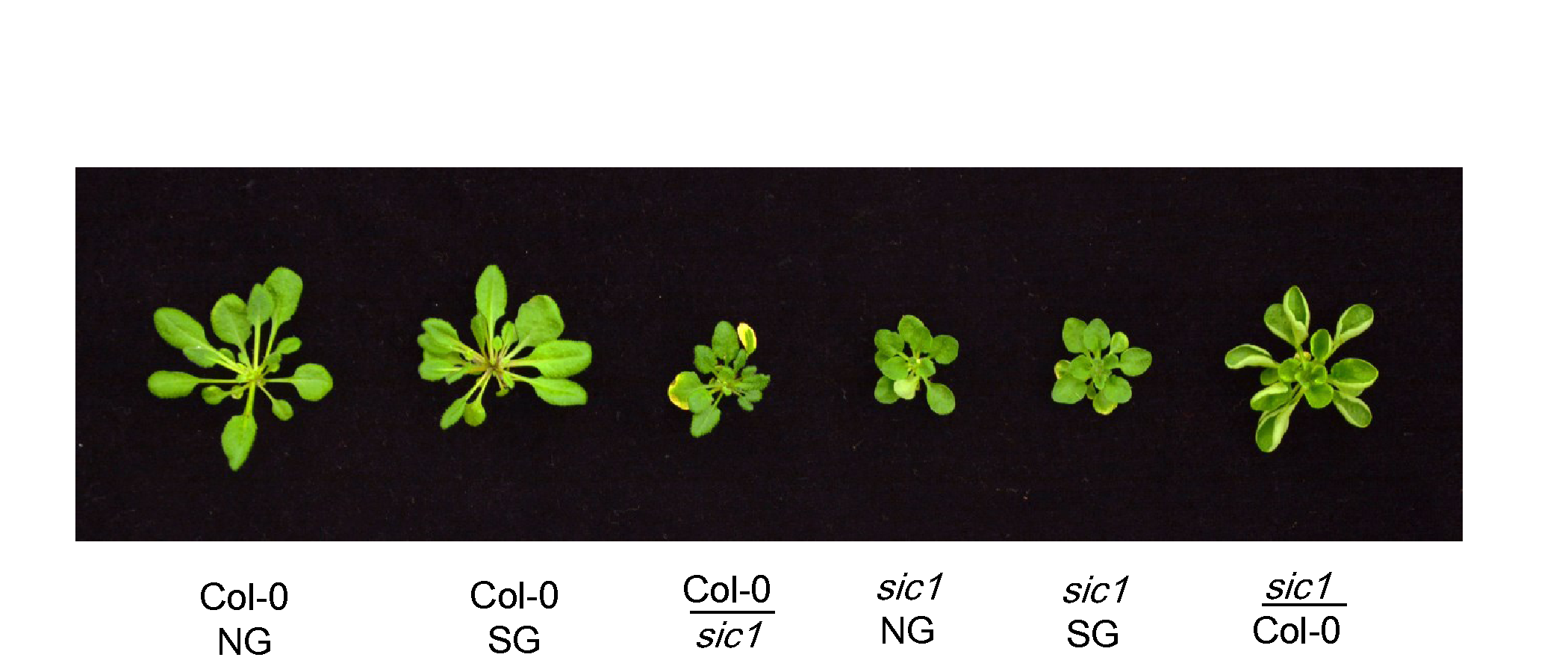

Supplement: S2 Fig — The images were taken on the 20th day after grafting. Col-0/sic1, grafted plants with Col-0 shoot and sic1 root; sic1/Col-0, grafted plants with sic1 shoot and Col-0 root. Col-0, Columbia-0; NG, non-grafted plants; SG, self-grafted plants; sic1, significant ionome changes 1. (TIF) [file pbio.2002978.s002.tif]

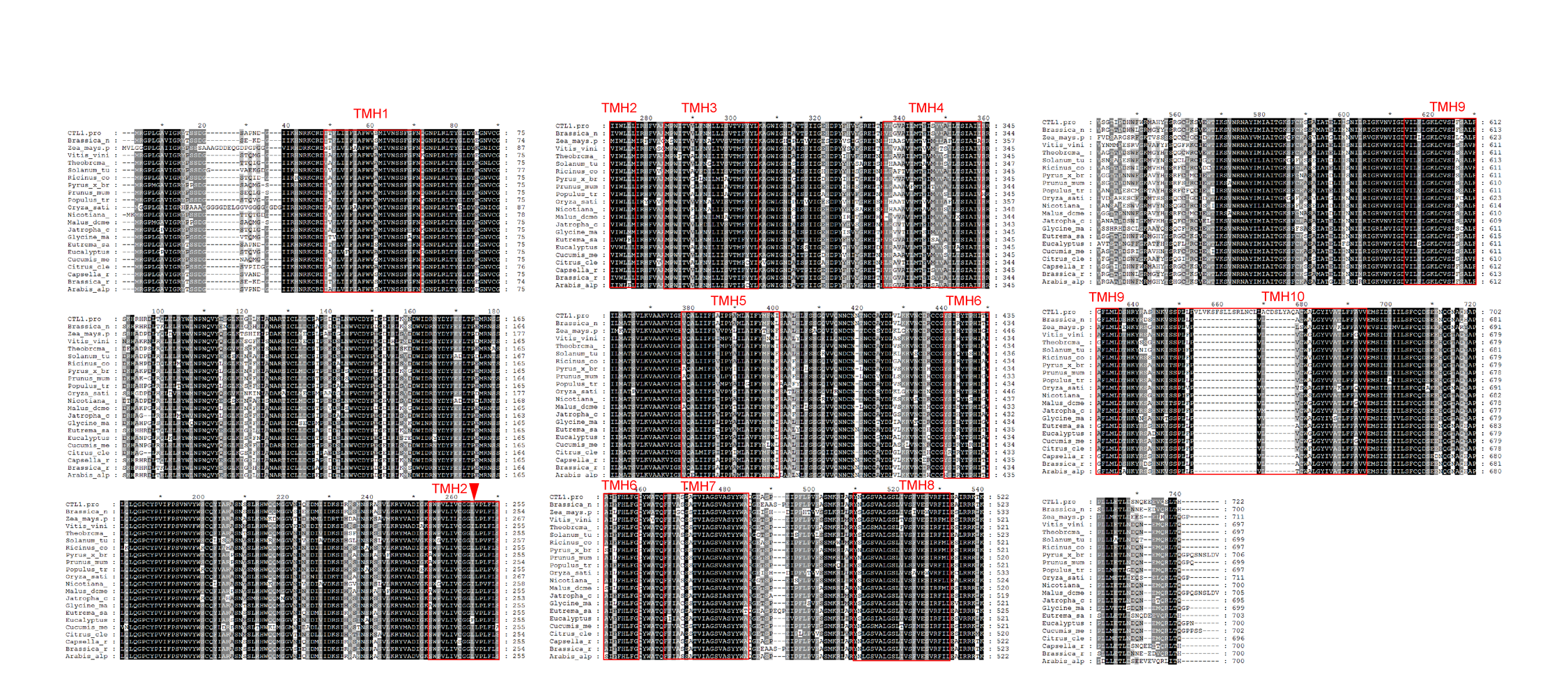

Supplement: S3 Fig — Protein sequence alignment of CTL1 among different species shows CTL1 is highly conserved and the mutation site of sic1 is located in a conserved domain. And the red boxes show the TMHs. The red triangle indicates the mutation site of sic1. CTL1, choline transporter-like 1; sic1, significant ionome changes 1; TMH, transmembrane helix. (TIF) [file pbio.2002978.s003.tif]

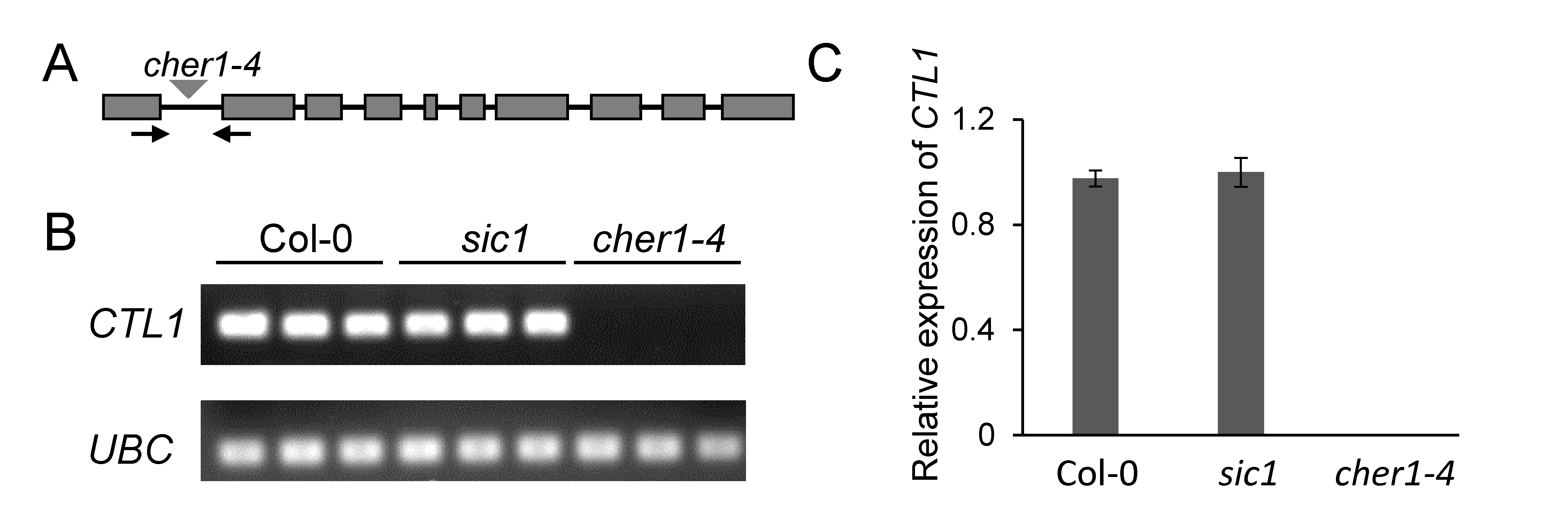

Supplement: S4 Fig — (A) The insertion site of cher1-4 and the position of primers used in this experiment. The triangle showed the insertion site of cher1-4 and the arrows shows the positions of primers used in this experiment (B) RT-PCR analysis of CTL1 transcripts in Col-0, sic1, and cher1-4. UBC was used as an internal standard for the RT-PCR. Single PCR reactions were performed on RNA from individual plants of each genotype. (C) The qRT-PCR result of CTL1 in Col-0, sic1 and cher1-4. The data represent the means ± SE; n = 3. The raw data can be found in S1 Data. Col-1, Columbia-0; CTL1, choline transporter-like 1; PCR, polymerase chain reaction; qRT-PCR, quantitative real-time polymerase chain reaction; RT-PCR, real-time polymerase chain reaction; sic1, significant ionome changes 1. (TIF) [file pbio.2002978.s004.tif]

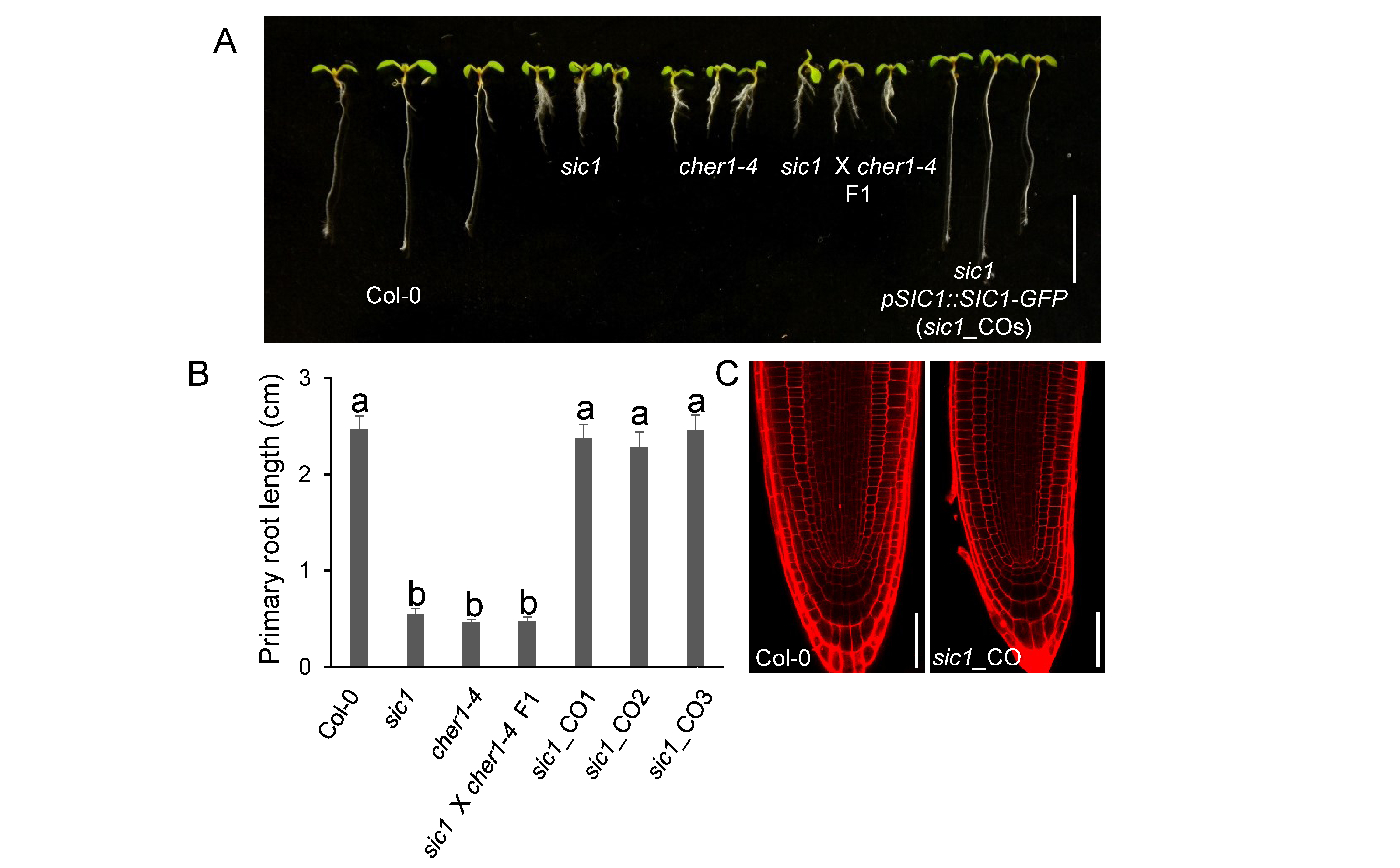

Supplement: S5 Fig — (A) The 6-day-old seedlings of Col-0, sic1, cher1-4, and complementation lines grown on agar-solidified 1/2 MS medium plate. Scale bar represents 1 cm. (B) Primary root length of Col-0, sic1, cher1-4, and complementation lines. Letters above bars indicate statistically different groups using a one-way ANOVA, followed by an LSD test at the probability of p < 0.05. Data represent means ± SE, n = 10 for each genotype. (C) Root patterning of Col-0 and sic1_CO plants. Scale bars represent 50 μm. The raw data can be found in S1 Data. Col-0, Columbia-0; LSD, least significant difference; sic1, significant ionome changes 1. (TIF) [file pbio.2002978.s005.tif]

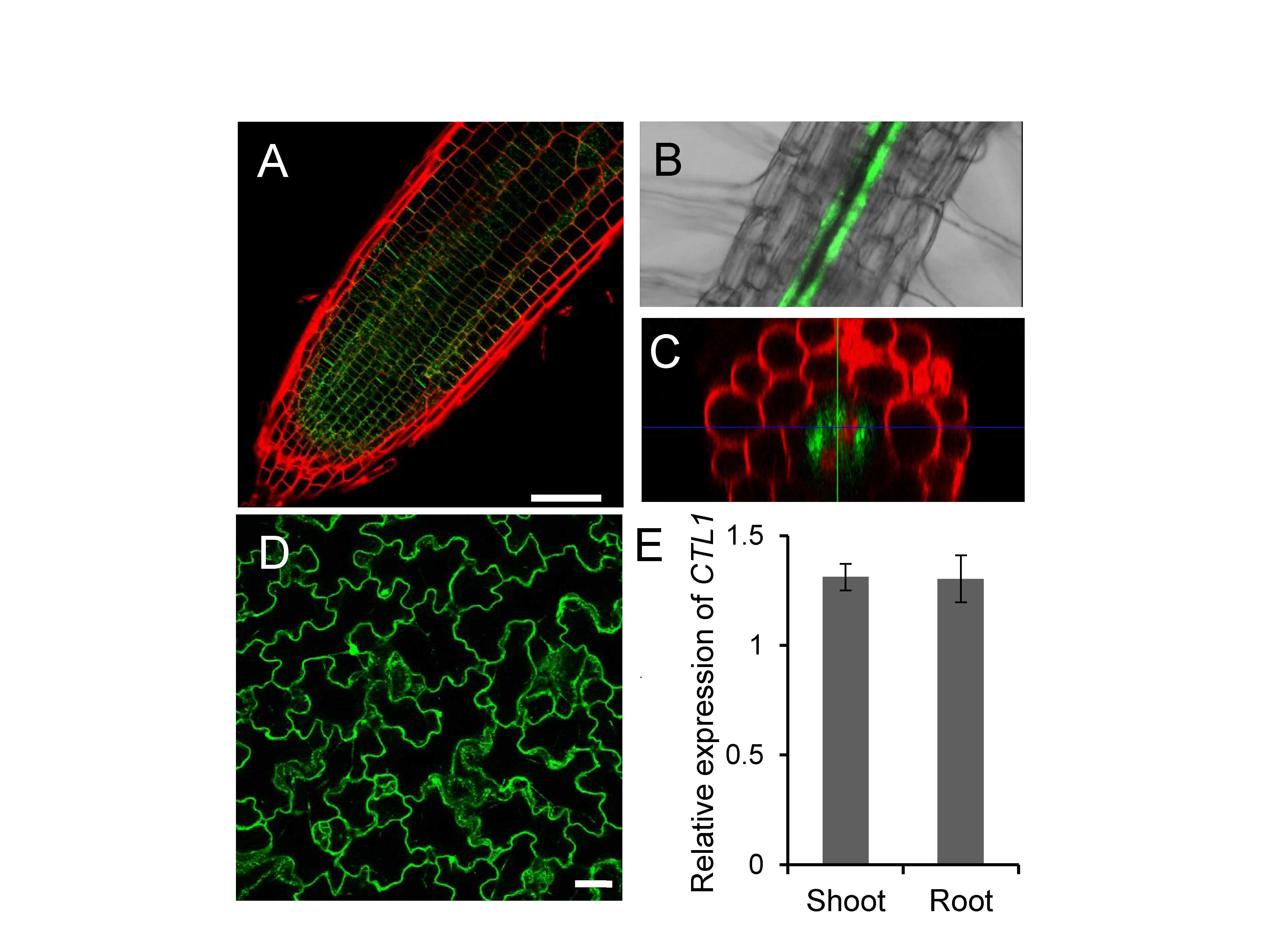

Supplement: S6 Fig — (A-D) CTL1-GFP showed the expression pattern in root tip (A), root maturation zone (B and C), leaf pavement cells (D). Three independent transgenic lines were observed and showed the same expression pattern. Green channel represents CTL1-GFP signal and red channel represents propidium iodide signal. Scale bars represent 50 μm for (A) and (D). (E) The relative expression of CTL1 in the shoot and root of Col-0 plants. Data represent means ± SE, n = 3. The raw data can be found in S1 Data. CTL1, choline transporter-like 1; GFP, green fluorescent protein. (TIF) [file pbio.2002978.s006.tif]

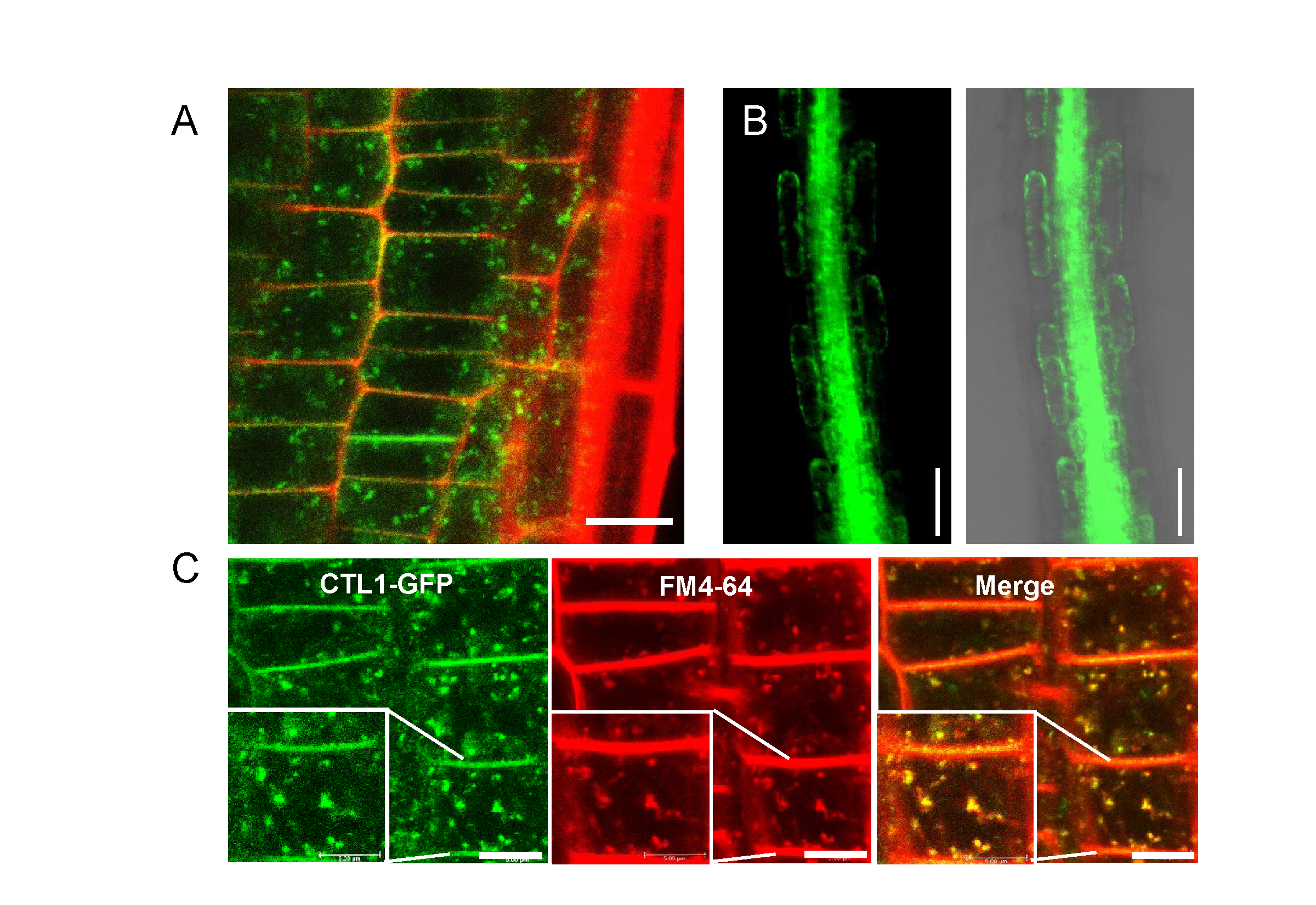

Supplement: S7 Fig — (A) An enlarged view of CTL1-GFP in the root tip of Col-0 plant. Green channel shows the GFP signal and red channel shows the PI signal. Scale bar represents 10 μm. (B) Subcellular localization of CTL1-GFP after plasmolysis in Col-0 root. Green channel shows the GFP signal. Scale bar represents 50 μm. (C) CTL1 is co-localized with FM4-64 in root cells. Green channel shows the CTL1-GFP signal; red channel shows the FM4-64 signal. The insets showed an enlarged view of epidermal cell. Scale bars represent 5 μm. Col-0, Columbia-0; CTL1, choline transporter-like 1; GFP, green fluorescent protein; FM4-64, N-(3-Triethylammoniumpropyl)-4-(6-(4-(Diethylamino) Phenyl) Hexatrienyl) Pyridinium Dibromide; PM, plasma membrane. (TIF) [file pbio.2002978.s007.tif]

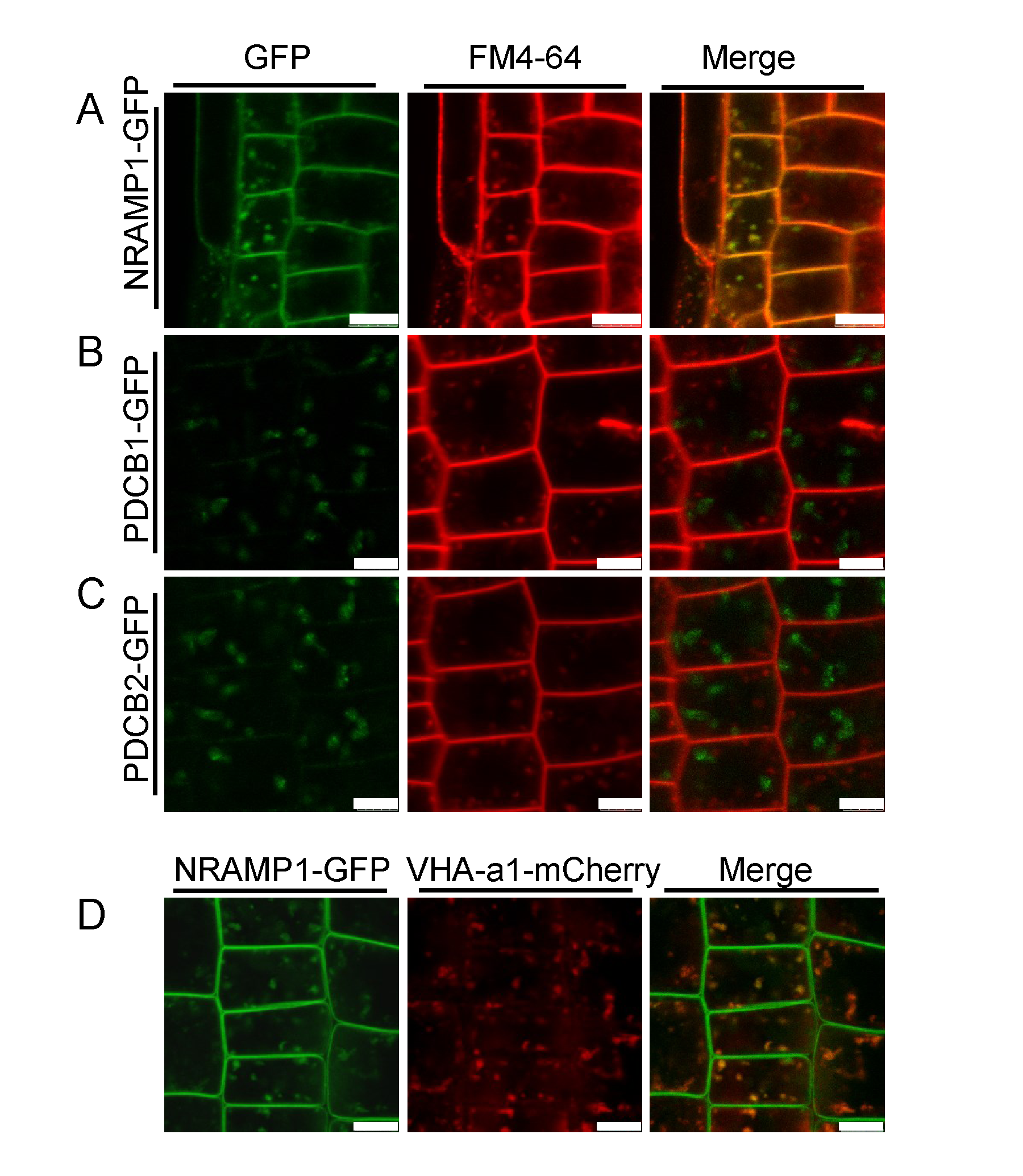

Supplement: S8 Fig — (A) The intracellular aggregation of NRAMP1-GFP is co-localized with FM4-64 signal in sic1. (B and C) The intracellular aggregations of PDCB1-GFP (B) and PDCB2-GFP (C) are not co-localized with FM4-64 in sic1. The transgenic seedlings of sic1 background were stained with FM4-64 for 1 h and then observed in confocal microscope. Green channels represent the GFP signal, and red channels represent the FM4-64 signal. The scale bars represent 7.5 μm in (A) and 5 μm in (B and C). (D) The intracellular aggregation of NRAMP1-GFP in sic1 localized to TGN. The scale bars represent 7.5 μm. Col-0, Columbia-0; CTL1, choline transporter-like 1; GFP, green fluorescent protein; FM4-64, N-(3-Triethylammoniumpropyl)-4-(6-(4-(diethylamino)phenyl)hexatrienyl)pyridinium dibromide; NRAMP1, natural resistance-associated macrophage protein 1; PDCB, plasmodesmata callose-binding protein; sic1, significant ionome changes 1. (TIF) [file pbio.2002978.s008.tif]

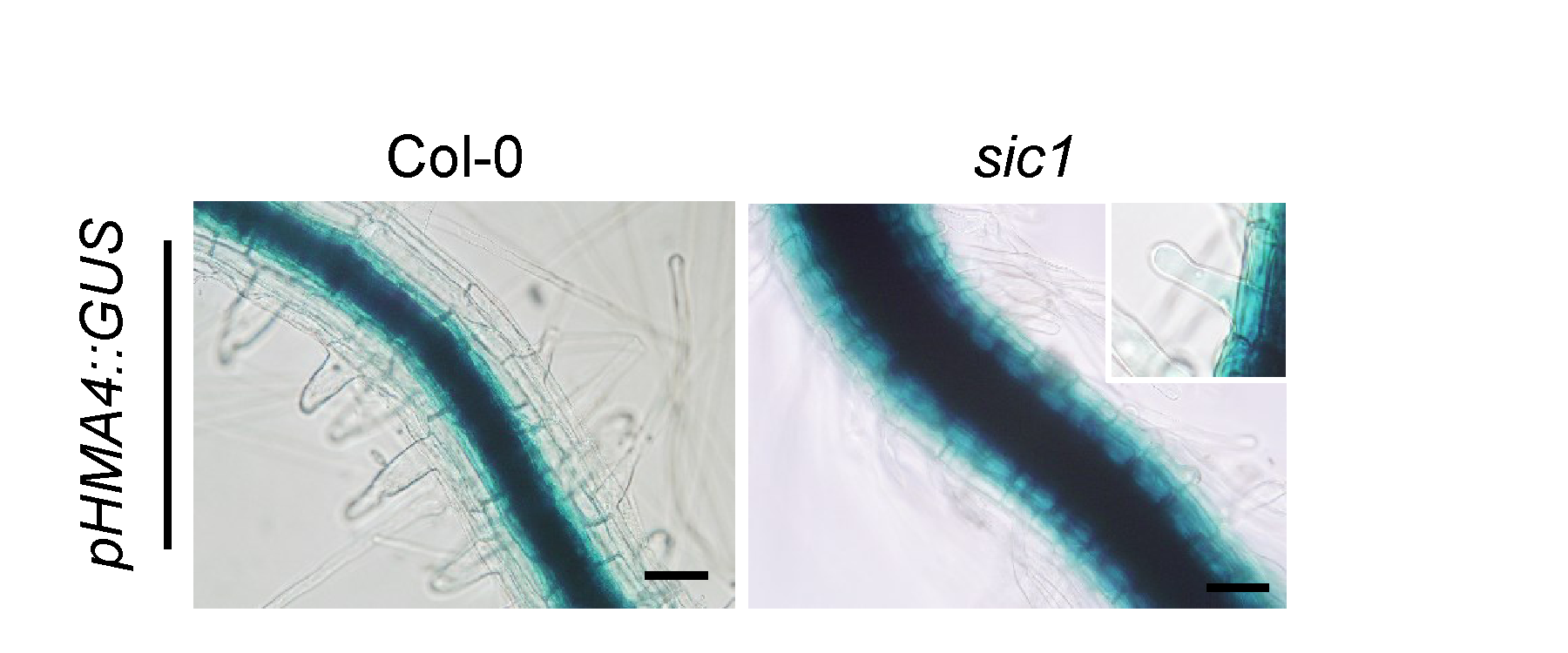

Supplement: S9 Fig — The insets showed an enlarged view of sic1 root hair. Scale bars represent 50 μm. Col-0, Columbia-0; HMA4, heavy metal ATPase 4; sic1, significant ionome changes 1. (TIF) [file pbio.2002978.s009.tif]

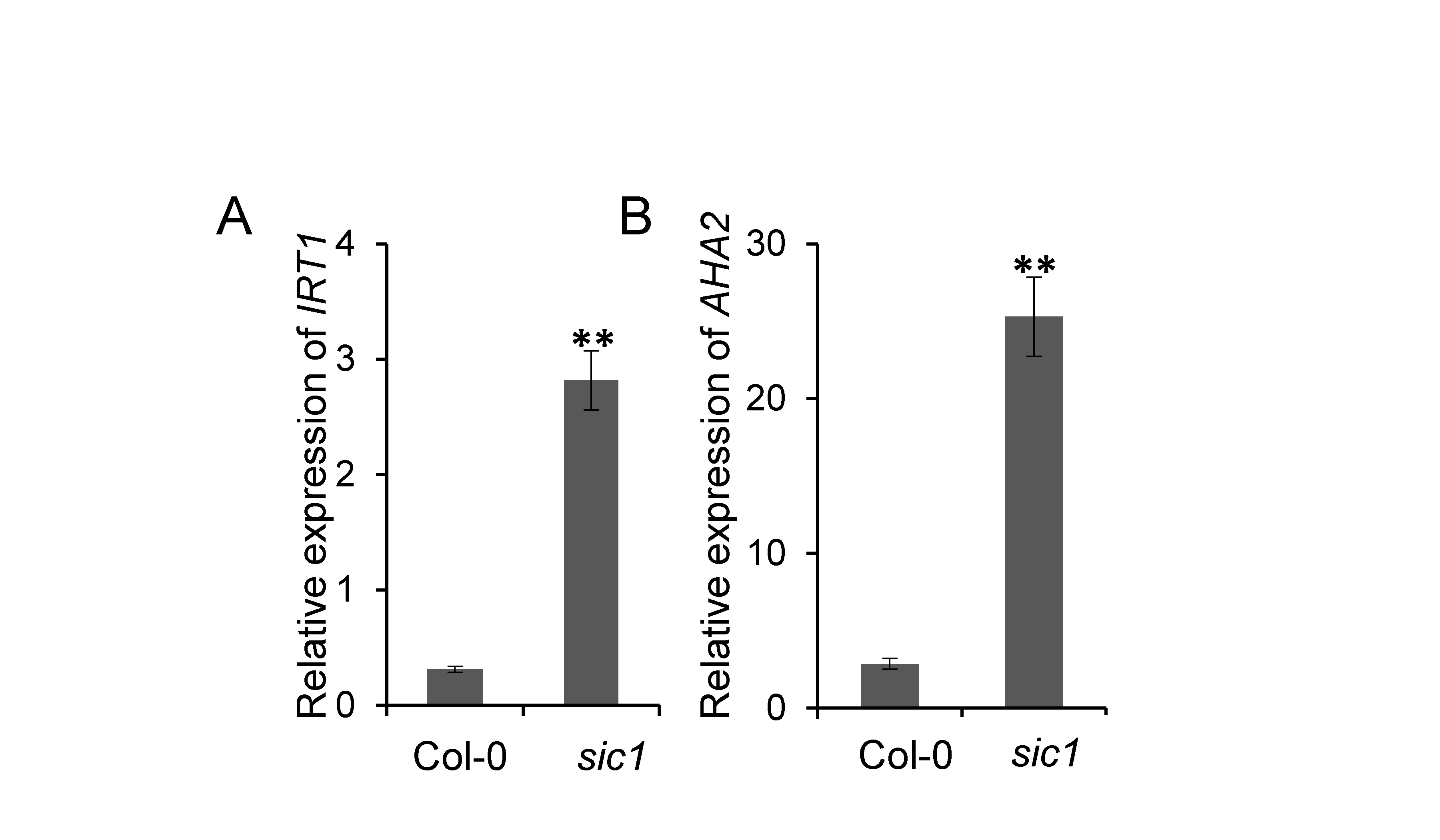

Supplement: S10 Fig — (A-B) The expression levels of IRT1 (A) and AHA2 (B) in the roots of Col-0 and sic1. The data represent the mean ± SE, n = 3. The asterisks above the bar represent a statistically significant difference (p < 0.01) calculated using Student t test. The raw data can be found in S1 Data. AHA2, Arabidopsis H +-ATPase 2; Col-0, Columbia-0; IRT1, iron regulated transporter 1; sic1, significant ionome changes 1. (TIF) [file pbio.2002978.s010.tif]

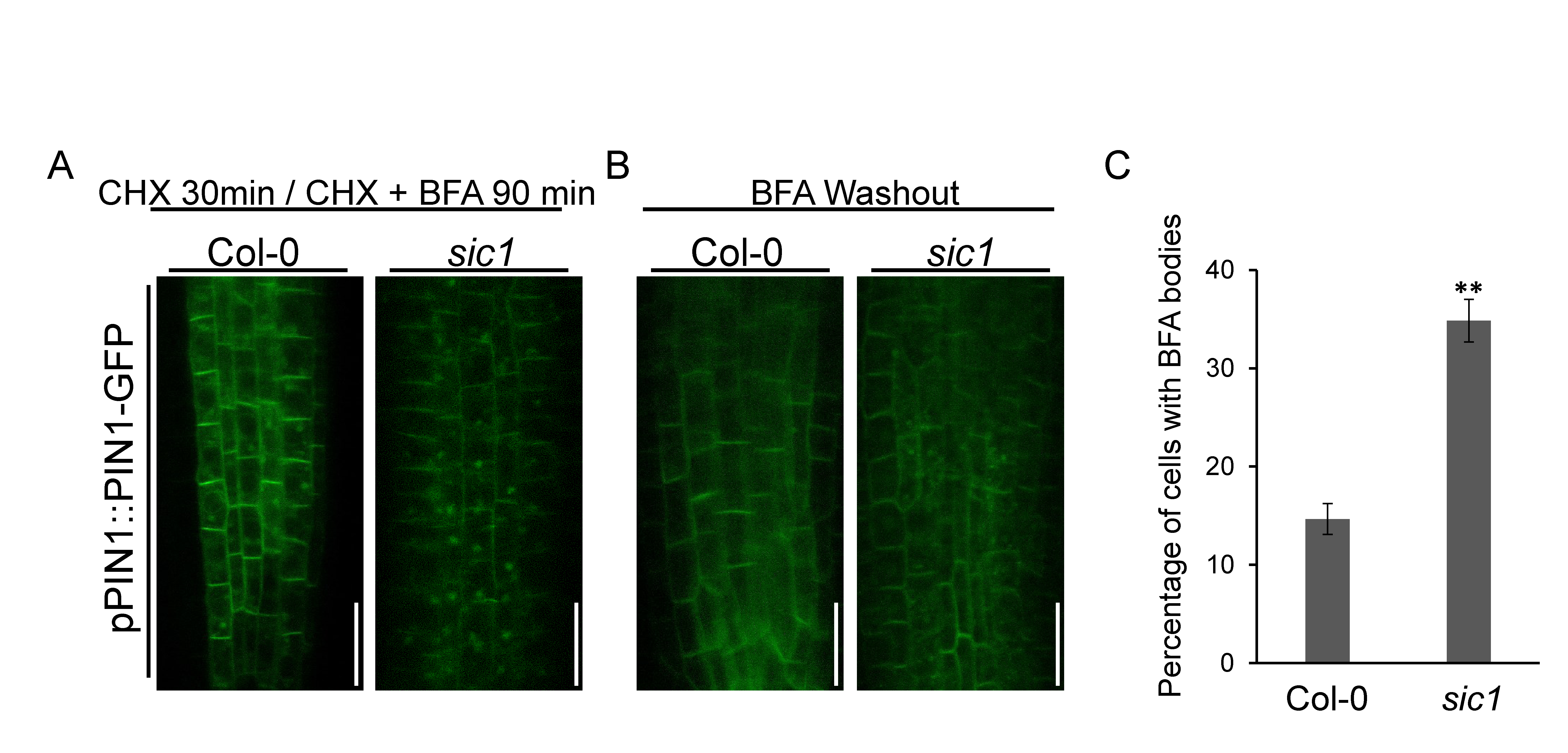

Supplement: S11 Fig — (A) The BFA compartments were observed after CHX and BFA treatment in Col-0 and sic1. (B) The subcellular localization of PIN1 in Col-0 and sic1 after BFA washout. Scale bars represent 20 μm. (C) The statistics analysis of percentage of cells with BFA bodies. The data represent the mean ± SE, ten seedlings of three independent transgenic lines were used in this analysis. The asterisks above the bar represent a statistically significant difference (p < 0.01) calculated using Student t test. The raw data can be found in S1 Data. BFA, brefeldin A; CHX, cycloheximide; Col-0, Columbia-0; PIN1, PIN formed 1; sic1, significant ionome changes 1. (TIF) [file pbio.2002978.s011.tif]
